# Biomarkers in Clinical Medicine Research: A Literature Survey in the PubMed Database and a Critical Evaluation

**DOI:** 10.3390/jcm15145518

**Published:** 2026-07-14

**Authors:** Dimitrios Tsikas, Katharina Habler, Stefan Ückert

**Affiliations:** 1 Core Unit Proteomics, Institute of Toxicology, Hannover Medical School, 30623 Hannover, Germany; 2 Institute of Laboratory Medicine, LMU University Hospital, LMU Munich, 81377 Munich, Germany; katharina.habler@med.uni-muenchen.de; 3 Department of Urology & Urological Oncology, Division of Surgery, Hannover Medical School, 30623 Hannover, Germany; streetgang@gmx.de

**Keywords:** analysis, biomarker, definition, database, disease, health, metabolites

## Abstract

Biomarker, the short form of “biological marker”, appeared in the scientific literature in the 1940s. Since then, many different definitions have been suggested, but a generally applicable explanation of the term biomarker in science is extremely challenging. The word biomarker is found in 1.3 million articles in the scientific database PubMed^®^ that currently comprises more than 39 million citations for biomedical literature. Biomarkers are closely associated with human health and disease. The present article attempts to approach and evaluate the multifaceted term “biomarker” from a clinical perspective by searching the PubMed database. The search term biomarker was combined with other search terms related to medicine, physiology, biochemistry, and chemistry. Currently generally accepted clinical biomarkers, such as the high-molecular-mass *N*-terminal prohormone of brain natriuretic peptide (NT-proBNP, 60%), prostate-specific antigen (PSA, 67%), and troponin (37%), serve as a kind of positive control. The combination of the search term biomarker with selected low-molecular substances of clinically non-validated and hence rather experimental character yielded surprisingly high fractions of 41% for 8-*iso*-prostaglandin F_2α_, 39% for symmetric dimethylarginine (SDMA), and 28% for asymmetric dimethylarginine (ADMA). The results of our survey are presented and discussed in detail for a wide spectrum of diseases. We focused on mechanisms that are assumed to underlie the biological activity and specificity of biomarkers. We also considered potential roles of the analytical chemistry of biomarkers including the emerging metabolomics and proteomics. Reliable analytical methods have been used for the quantification of the isomeric low-molecular-mass ADMA and SDMA in human biological samples. ADMA, but not SDMA, is considered an endogenous inhibitor of the endothelium-derived nitric oxide (NO) synthesis, one of the most potent endogenous vasodilators. Paradoxically, the utility of ADMA and SDMA as biomarkers in the renal and cardiovascular systems seems to contradict their main biological activity. This prominent pair is representative of many biomarkers and reveals that the supposed biomarker utility is likely to be predicated on not yet considered biological activity. The majority of human diseases are heterogenic, affect many organs and seem to include different and overlapping biochemical pathways. In recent years, especially proteomic studies provided a series of new potential candidate biomarkers. However, such biomarkers must still be validated in the clinic before they can be introduced into clinical practice. This is perhaps the most critical phase in the discovery of disease biomarkers. Our analysis reveals that the area of biomarker research is highly challenging. With minor exceptions, there is no specific biomarker for a single disease. In addition to clinical examinations, a combination of several biomarkers seems to be needed for reliable diagnosis and therapy. Analytical chemistry, especially proteomics, delivers a huge amount of data, which may complicate and even hinder progress in this area. Specific quantitative analysis of candidate biomarkers observed by proteomics (and metabolomics) is highly recommended to proceed with the same biological samples from studies in which the biomarkers were discovered.

## 1. Introduction

The word “biomarker” is the short form of “biological marker” and synonymous with “biochemical parameter”. The Greek word bios (βίος) means life. The term “biological marker” was introduced in the 1940s in the scientific literature. Since then, many different definitions have been provided for biomarkers and several review articles have dealt with various aspects of this fundamentally important issue [1,2,3,4,5]. Significant confusion persists regarding the use of fundamental definitions and concepts, both in research and in clinical practice. Definitions of the term biomarker were proposed by the U.S. Food and Drug Administration (FDA) and by the National Institutes of Health (NIH). They were reviewed by Puntmann [2] and more recently by Califf [3].

Depledge [1] defined a biomarker in the specific area of Earth’s marine ecosystems as follows:

*A biochemical, cellular, physiological or behavioral change which can be measured in body tissues or fluids or at the level of the whole organism that reveals the exposure at/or the effects of one or more chemical pollutants.*

According to the National Institutes of Health [2], a biomarker is as follows:

*A characteristic that can be objectively measured and evaluated as an indicator of normal biological processes, pathogenic processes or pharmacological responses to a therapeutic intervention*.

In the present article, the term biomarker is synonymous with the following:

*A low-molecular-mass (inorganic or organic) or high-molecular mass chemical substance that is ubiquitously present in biological samples (e.g., blood, urine, CSF, tissue), is accessible to quantitative analysis, and its quantity (e.g., concentration, content) serves to define the status of a biological system (i.e., healthy, disease) in cells, organs, or in the whole human organism.*

The NIH and the FDA proposed a set of definitions that should guide researchers in developing needed evidence and practitioners in the application of biomarkers in health care [3]. These organizations suggested approaches to biomarker development that incorporate collaborative regulatory science involving multiple disciplines. NIH and FDA also emphasized the necessity to ensure that rational, evidence-based biomarker development keeps pace with scientific and clinical needs [3].

A single biomarker may meet multiple criteria for different uses. Biomarker definitions may overlap, but they also have clear distinguishing features that specify particular uses. A number of subtypes of biomarkers have been defined according to their putative applications. Califf [3] distinguished and explained the following subtypes of biomarkers:

(1) Diagnostic;
(2) Monitoring;
(3) Pharmacodynamic/response;
(4) Predictive;
(5) Prognostic.

**Figure 1:**
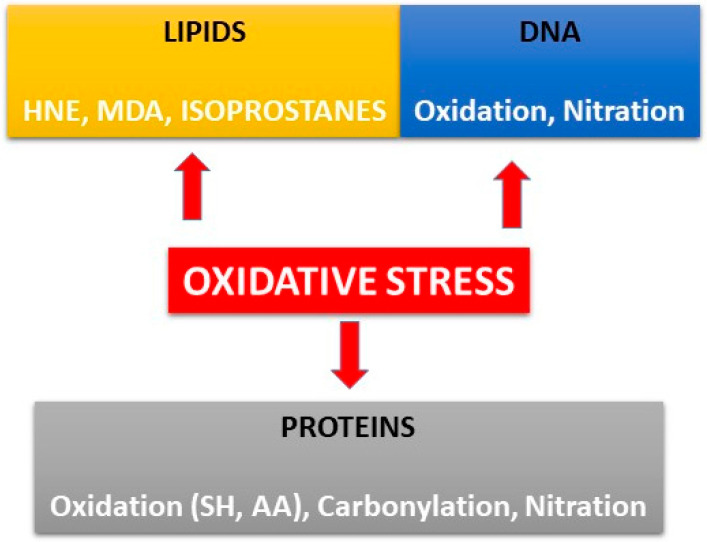
Schematic of widely used biomarkers of oxidative stress. HNE, 4-Hydroxy-nonenal; MDA, malondialdehyde; AA, amino acid. SH, sulfhydryl. Modified after Giustarini et al. 2009 [5].

Biomarkers relating to oxidative stress are another subtype that has not been considered by Puntmann [2] and Califf [3]. The groups by Dalle-Donne and Rossi [4,5] discussed the biomarkers of oxidative stress in human disease in relation to the chemical class of oxidized biomolecules. They include lipids, proteins, amino acids, and DNA, which can be of cellular and/or extracellular origin (Figure 1). Among other issues, the authors addressed aspects of the choice of the biomarker and the biological system, the use of antioxidants in clinical studies, and factors that can influence the outcome of treatments/supplementation with antioxidants. In their analysis, Giustarini et al. [5] observed that as many as 71 different biomarkers of oxidative stress have been measured but not adequately validated. The authors proposed a set of characteristics and requirements for biomarkers of oxidative stress in humans. They include [5]:

(a) Chemical stability;
(b) Direct implication in the onset and/or progression of disease;
(c) Non-invasive assessment by validated quantitative analytical methods;
(d) Low intra- and inter-variability;
(e) Consensus and establishment of reference intervals and values.

The present work undertakes an attempt to approach the multifaceted term biomarker by analyzing the scientific literature archived in the database PubMed. PubMed^®^ is an official website of the United States government. This database currently comprises more than 39 million articles of scientific biomedical literature from MEDLINE, life science journals, and online books. It is a freely available (https://pubmed.ncbi.nlm.nih.gov/, accessed date 2 January 2026), consistently updated and useful source of reviewed scientific work in medicine and affiliated disciplines including physiology, biochemistry, chemistry and physics.

It is not surprising that the words human, disease and health occur very often in PubMed, with some articles dating back to the 18th century (Table 1).

“Marker” and “biomarker” belong to the most frequently occurring words in PubMed. By using the search terms “marker” and “biomarker” more than 1 million articles (about 3% of total) are found in PubMed. The articles, in which “marker” and “biomarker” occurred, go back to the years 1928 and 1946, respectively. In the survey of this article, the search term biomarker is used. The general term “biomarker” needs specification. Thus, the use of combinations of the term biomarker with other general terms such as “disease” resulted in 585,595 articles accounting for a fraction of 1.5% of the total.

## 2. Methods

The PubMed data bank can be searched in many ways. In the present survey, we used the “Advanced Search Builder” for individual search terms or combinations of search terms within all parts of the “All Fields” of the articles. No inclusion or exclusion criteria were used within the PubMed database literature search. It should be emphasized that such searches are expected to be “raw”. Because of its complexity due to the consideration of biomarkers from many different diseases and many different analytical methodologies, the present survey is not a meta-analysis or a systematic review. Thus, we did not present workflows of the performed searches here, which are usually presented in meta-analysis and systematic review articles. The PubMed software provides a list of articles found by the search and a figure. The data can be exported (.csv) and further analyzed graphically or in the form of tables as performed in the present work.

To gain more specific information on the use of the term biomarker in the biomedical sciences, the term biomarker was used as the search term 1 in combination with various other specific terms, i.e., search term 2. For instance, the search term 2 was a specific disease (e.g., cancer, diabetes), an organ or fluid of the human body (e.g., kidney, blood), or a chemical substance of endogenous (e.g., creatinine) or exogenous (e.g., drugs) origin. The percentage portion of the number of articles obtained by the combination of the search terms 1 and 2 relative to the number of articles obtained by the search term 2 was used as a first rough estimate of the specificity of the respective biomarker. As an example, the search term 2 prostate yielded 300,726 hits. Of these articles, 55,162 articles were found to be associated with the search term 1 biomarker. This corresponds to a fraction of 18.3% of term 2 (Table 2). The first article that associated the prostate with a biomarker appeared in 1972. In our opinion, such information is valuable and was included in the results presented in the tables as the year of first mention. We therefore did not use any search filters for the period. In many cases, the year of onset of yearly published articles may also be of interest and was included in the tables.

In this context, it is considered that the higher the fractional value the higher the suitability of the biomarker for a certain disease ascribed by scientists. The numbers reported in the present article observed by searching PubMed are in no way appropriate to evaluate the clinical significance of the biomarkers.

The results of the survey of the literature found in PubMed were assessed early in 2026. They are presented, interpreted and discussed in Section 3 and Section 4. In Section 3, the results are presented and discussed from a general scope. In Section 4, the results of the survey are presented from a more specific scope such as the involvement of particular biochemical pathways or oxidative stress. We wish to emphasize that our observations from the PubMed search should be considered as a first estimate. They do not have the potential for far-reaching conclusions. We considered and used them as a starting point for deeper examination by including additional aspects.

## 3. General Results and Discussion

### 3.1. Biomarker and Organs, Body Fluids and Tissues of the Human Body

The prostate is an accessory gland of the male reproductive system. The term prostate has been found since 1972 in PubMed in a relatively small number of articles, but with 18%, it possesses the highest ranking among the organs searched.

The combination of “biomarker” with a biological fluid resulted in fractions between 4.4% and 11.7% (see Table 3). Table 3 indicates that among the body fluids searched, saliva possesses the highest ranking (11.3%), followed by urine (10.3%) and blood (9.5%) in the context of biomarker.

### 3.2. Biomarker and Human Diseases or Syndromes

The combination of “biomarker” with a specific disease resulted in fractions of 12.6% for “Alzheimer”, 6.3% for “Parkinson”, 10% for “cancer”, and 6.3% for “diabetes” (see Table 4). Thus, Alzheimer’s disease has the highest ranking among the diseases and syndromes followed by cancer, although the number in comparison to cancer-related articles is only 1/20th (Table 4).

Table 5 indicates that among *established* biomarkers with clinical relevance, i.e., creatinine, troponin, *N*-terminal prohormone of brain natriuretic peptide (NT-proBNP), and prostate-specific antigen (PSA), the two latter achieved the highest ranking among the category biomarkers, i.e., NT-proBNP (67%) and PSA (60.0%). Despite the small number of articles, 8-*iso*-prostaglandin F_2α_, which is considered a biomarker of oxidative stress (lipid peroxidation) [4,5], occupies the third highest ranking.

Table 6 summarizes the results of the search term 1 *biomarker* and the substance class of (A) amino acids and some of their metabolites and (B) biogenic amines and polyamines. Interestingly, ADMA and SDMA achieved the highest rankings among these classes.

The combination of search term 1 *biomarker* with various additional classes of substances resulted in the highest rankings for microRNA (23.9%) and the albumin ratio (24.6%) (Table 7).

Amino acid residues in proteins and other biomolecules undergo numerous enzymatic and non-enzymatic post-translational modifications (PTMs). The results of the combination of the search term 1 *biomarker* with the search term 2 post-translational modification are listed in Table 8. The highest rankings were observed for citrullination (17.7%) and DNA methylation (17.9%).

## 4. Specific Results and Discussion

In theory, each low-molecular-mass (LMM) and each high-molecular-mass (HMM) natural or synthetic substance (including drugs and toxins) has the potential to function as a biomarker (see the Tables in Section 3). LMM biomarkers such as malondialdehyde (MDA, 12%), total homocysteine (hCys, 20%), ADMA (27.6%), and SDMA (38.6%) have relatively high percentage fractions. HMM substances such as troponin (36.8%), NT-proBNP (60%) or PSA (66.5%) have partly considerably higher percentage fractions. What do these numbers tell us? Does such a literature search result in meaningful and usable information about biomarkers in science in general and in clinical medicine in particular, which takes care of human health? This issue is discussed in the following paragraphs on selected examples including metabolic pathways, and the origin of certain biomarkers.

Generally, the concentration of LMM and HMM substances in biological samples of humans varies in certain ranges even if measured therein under standardized conditions such as overnight fasting, no diet and no supplementation. Concentration ranges that are determined in health and disease generally overlap to a considerable degree. This represents a formidable challenge in the classification of health status as “healthy” or “diseased”, which is crucial for subsequent medical decisions and actions such as pharmacotherapy and even surgery. Needless to say, the values and intervals of biomarkers may depend upon age, gender, lifestyle (alcohol, smoking) and even ethnicity. For instance, studies suggest that high plasma or serum concentrations of ADMA are considered biomarkers of cardiovascular disease in adults but not in children (see below). Biomarkers not only serve as qualitative indicators in the diagnosis of disease. Their magnitude, i.e., their concentration measured in biological samples may serve as a quantitative measure to grade the severity of certain diseases such as in the case of PSA (see below). Low circulating concentrations of some vitamin substances such as vitamin D and vitamin C (ascorbic acid) may also serve as biomarkers for certain diseases. Thus, both high and low concentrations of biomarkers may indicate altered health status and the need to undertake proper measures such as supplementation.

It should be noted that some biomarkers such as creatinine may have different utilities in human life and in the sciences. Creatinine in serum (plasma) is an indicator and measure of kidney function, especially of its glomerular filtration rate, with higher concentrations indicating worse kidney function. On the other hand, creatinine in urine is a useful parameter for the correction of the excretion rate of both LMM and HMM substances measured in urine when it is collected by spontaneous micturition, which is common in many clinical studies and in clinical diagnosis (albumin/creatinine ratio).

### 4.1. The L-Arginine/Nitric Oxide Pathway

Nitric oxide (NO) and L-citrulline are endogenously produced from L-arginine by nitric oxide synthase (NOS) isoforms [6]. The L-arginine/NO pathway consists of numerous metabolites, which derive from L-arginine and L-citrulline on the one hand and from NO on the other hand. Major NO metabolites are nitrite and nitrate, which circulate in the blood and are excreted in the urine. From a clinical perspective, the most important L-arginine-derived amino acids include asymmetric dimethylarginine (ADMA) and symmetric dimethylarginine (SDMA). They stem from the PTM of L-arginine residues in numerous largely unknown proteins. ADMA and SDMA occur both in their free forms and in proteins as residues.

Free ADMA is known to be a weak inhibitor of NO biosynthesis from L-arginine catalyzed by the constitutively expressed endothelial nitric oxide synthase (eNOS) [6]. In general, SDMA is not considered an inhibitor of NO biosynthesis. The biological activity of ADMA- and SDMA-proteins is still unexplored. NO is one of the strongest endogenous vasodilators and inhibitors of platelet aggregation. Free ADMA and SDMA are released from their proteins by regular proteolysis. Both ADMA and SDMA are considered biomarkers of renal and cardiovascular diseases. It is assumed that the inhibition by ADMA of the NO biosynthesis in the endothelial cells qualifies ADMA as a biomarker. Which biological activity of SDMA may explain the function of SDMA as a biomarker is still unknown. ADMA and SDMA proteins have been rarely measured in blood but have not been explicitly investigated as biomarkers so far. Thus, ADMA and SDMA represent an example of biomarkers, of which the basic principle that defines their biomarker eligibility is unknown or uncertain [7].

Dimethylaminine (DMA) is the major circulating and urinary metabolite of ADMA. Urinary DMA is considered an index parameter of whole-body ADMA synthesis [8]. SDMA is excreted in the urine almost nonmetabolized and is used by some scientists as a measure of kidney function.

Nitrite and nitrate have been used in many studies as measures of systemic (nitrite) and whole-body (nitrate) synthesis of L-arginine-derived NO in humans [6].

Exogenous nitrite, nitrate and DMA from food and drinking water, but not ADMA or SDMA, can considerably contribute to endogenously produced nitrite, nitrate and DMA and this limits their appropriateness as biomarkers. Note that exogenous nitrite and nitrate are considered cancerogenic and toxic due to their potential to form nitrosamines and methemoglobin, respectively.

### 4.2. Eicosanoids—Arachidonic Acid Pathways

Eicosanoids are derived from the C_20_-polyunsaturated fatty acid arachidonic acid and include the primary prostaglandins, thromboxane, leukotrienes, and their metabolites. They circulate in the blood and are excreted in the urine. Primary eicosanoids possess chemical reactivity and are biologically highly potent in various organs, while most of their metabolites lack biological activity. Both primary eicosanoids and their index metabolites serve as indicators of the synthesis of eicosanoids in certain cells such as endothelial cells (prostacyclin) and platelets (thromboxane), or in organs such as the kidney (prostaglandin E_2_). Urinary excretion rates of index metabolites are commonly used as measures of whole-body synthesis [9]. 8-*iso*-Prostaglandin F_2α_ occurs in blood as a free acid and esterified to lipids and as a free acid and glucuronide in the urine [9]. Eicosanoids have been associated with numerous diseases. Occasionally, some eicosanoids were found at elevated urinary excretion rates such as Bartter’s syndrome [10,11] and hyper-prostaglandin syndrome (HPS) [12]. Both pathological conditions are associated with stimulated prostaglandin E_2_ synthesis, or due to inherited peroxisomal beta-oxidation deficiency in the Zellweger syndrome [13,14].

Among the eicosanoids, the 8-*iso*-prostaglandin F_2α_ has with 41% the greatest fraction (Table 5). 8-*iso*-Prostaglandin F_2α_ is one of maximally 64 F_2_-prostaglandins, which are believed to form mainly non-enzymatically and are therefore generally considered as biomarkers of oxidative stress, namely of lipid peroxidation [12,15]. A specific disease is not assigned to 8-*iso*-prostaglandin F_2α_, but various diseases are thought to be associated with elevated oxidative stress (lipid peroxidation). Urinary 8-*iso*-prostaglandin F_2α_ has been reported to be a biomarker in addition to known risk factors of coronary heart disease, i.e., diabetes mellitus, hypercholesterolemia, hypertension, and smoking, and to be correlated with the number of risk factors [16].

### 4.3. Malondialdehyde and Total Homocysteine

Malondialdehyde (MDA), 4-hydroxynonenal and related aldehydes are products of lipid peroxidation of polyunsaturated fatty acids, including arachidonic acid, and possess chemical reactivity and cytotoxicity [17]. MDA is the most widely used biomarker of oxidative stress [18]. Interestingly, the literature search in the present article revealed a fraction of 12% for MDA but 40% for 8-*iso*-prostaglandin F_2α_, although the number of reported articles of MDA is 40 times higher (Table 5). One could assume that low numbers of articles can produce bias. Like 8-*iso*-prostaglandin F_2α_, MDA is generated both by enzymatic and non-enzymatic reactions, can be artefactually formed during the storage of lipid-rich biological samples such as plasma even at low temperatures, and cannot be assigned to a specific disease. In contrast to 8-*iso*-prostaglandin F_2α_, MDA is preferentially analyzed in plasma or serum and occurs at much higher concentrations (µM vs. pM).

In a large cohort of renal transplant recipients (RTRs; *n* = 604), circulating MDA concentration was found to be independently associated with long-term risk of cardiovascular mortality, particularly in RTRs with relatively lower ascorbic acid concentrations or renal function [19].

The amino acid homocysteine occurs in blood as a free acid and as an asymmetric disulfide with albumin and other proteins. Total plasma homocysteine, with a fraction of 20% (Table 5), has been widely used as a biomarker of oxidative stress with clinical relevance and as an independent risk factor in over 100 conditions, notably atherosclerosis [4,5]. Plasma total homocysteine has been proposed as a guide for the prevention of disease [20]. Total homocysteine has been thoroughly used in epidemiological and clinical studies as a measure of the effects of antioxidants including various vitamins on the concentration of total homocysteine and diseases. The outcome of prospective, randomized clinical trials remained largely inconclusive [4,5,21]. Obviously, scientists have set great expectations for the use of antioxidants in clinical studies. An explanation for the great disappointments could be that their hypotheses were based on observations from in vitro experiments and animal studies, in which high concentrations or doses of antioxidants had been used that were higher than those used in humans [22].

### 4.4. Amino Acids, Biogenic Amines and Polyamines

Among the non-metabolized α-amino acids, the non-proteinogenic citrulline (17.6%) has by far the highest fraction (Table 6), to which the PTM citrullination (17.7%) and citrullinemia (5.8%), an autosomal recessive urea cycle disorder, are likely to be the major contributors. Tyrosine (8.3%) and alanine (8.1%) have similar fractions. The biogenic amines, polyamines and catecholamines have relatively low fraction values (Table 6). Interestingly, arginine has a low fraction value, yet its PTM by *N*^G^-methylation (to ADMA and SDMA) and *N*^G^-citrullination (to citrulline) resulted in much higher values (Table 8).

Nitration of amino acid residues in proteins, notably of tyrosine to 3-nitrotyrosine due to its higher chemical reactivity because of its phenolic group, is not catalyzed by enzymes. The PubMed search yields a surprisingly high fraction of 15.3% (Table 8). Many scientists consider 3-nitrotyrosine a biomarker of oxidative (nitrative) stress. It is worthy of mention that this is due to the use of questionable commercially available immunological and immunohistochemical assays. Accurate and artifact-free quantitative analysis by mass spectrometry-based methods, such as GC-MS/MS and LC-MS/MS, questions the utility of 3-nitrotyrosine as a biomarker of nitrative stress. The application of sophisticated mass spectrometric techniques is advantageous and essential [23,24].

In the review by Daiber and colleagues from the year 2021 [25], human and animal studies were discussed regarding associations of oxidative stress biomarkers with cardiovascular disease/markers. With respect to 3-nitrotyrosine three studies in humans and two studies in animals were considered. In the animal studies, 3-nitrotyrosine had been analyzed by immunohistochemistry and dot plot in rat hearts and was found to be increased after ischemia/reperfusion injury and after myocardial infarction. In the human case–control study on 100 CAD patients and 108 controls, 3-nitrotyrosine residues in plasma proteins had been quantified by stable-isotope dilution ion trap LC-ESI-MS/MS [26]; increased 3-nitrotyrosine concentrations had been measured in the CAD patients. In the prospective cohort study on 342 CAD patients, 3-nitrotyrosine had been analyzed in serum by colorimetric ELISA [27]; no relationship had been found between 3-nitrotyrosine and mortality rates during a 4-year follow-up. In the clinical trial on 120 pediatric patients, 3-nitrotyrosine had been analyzed in plasma by ELISA [28]; after 75 g oral glucose tolerance tests, 3-nitrotyrosine had been found to be related to CAD. Below, we briefly discuss in more detail the study by Shishehbor et al. [26] from the year 2003 who used LC-MS/MS for the measurement of 3-nitrotyrosine.

Shishehbor et al. [26] reported that nitrotyrosine levels were higher in patients with CAD compared with controls (median values, 9.1 µmol/mol tyrosine vs. 5.2 µmol/mol tyrosine, respectively; *p* < 0.001). Patients in the highest quartile of nitrotyrosine levels had an increased risk of CAD compared with patients in the lowest quartile. Nitrotyrosine levels correlated with age (r = 0.14, *p* = 0.03), fasting triglycerides (r = 0.14, *p* = 0.03), and CRP levels (r = 0.15, *p* = 0.02); however, these associations are very small in magnitude and account for less than 5% of the observed variance in nitrotyrosine. There was no significant correlation between nitrotyrosine and LDL-C, HDL-C, or total cholesterol. Participants with diabetes had higher nitrotyrosine levels than those who did not have diabetes (median values, 9.6 µmol/mol tyrosine vs. 5.7 µmol/mol tyrosine, respectively; *p* < 0.001).

Shishehbor et al. [26] used in their study an LC-ESI-MS/MS method previously reported by Brennan et al. [29], which had been originally developed for the measurement of 3-nitrotyrosine in proteins recovered in the supernatant of peritoneal lavage from mice. Neither Shishehbor et al. [26] nor Brennan et al. [29] reported on the analytical performance of the LC-MS/MS method for 3-nitrotyrosine. The 3-nitrotyrosine/tyrosine values in human plasma measured by GC-MS/MS are 5 to 10 times lower and much less variable [24] compared to LC-MS/MS [26,29].

3-Nitrotyrosine measurement in biological samples demands expert and attentive mass spectrometrists who are committed to developing and applying thoroughly validated analytical methods. Until the present day there is no convincing evidence by GC-MS/MS, LC-MS/MS or proteomics that 3-nitrotyrosine is a dependable clinical biomarker [24].

**Figure 2:**
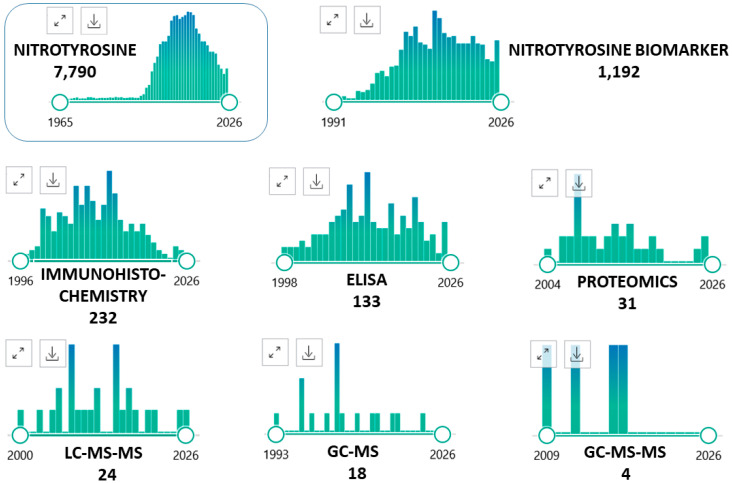
Number of articles found in PubMed by using the search term “nitrotyrosine” and “nitrotyrosine biomarker” (upper panel) and by using the combination of “biomarker nitrotyrosine” with “immunohistochemistry”, “ELISA”, “proteomics”, “LC-MS/MS”, “GC-MS” and “GC-MS/MS”.

A possible explanation of the unexpectedly high percentage fraction of 15.3% for nitrotyrosine could be the extremely low number of articles found in PubMed by using the combination of “biomarker nitrotyrosine” with “immunohistochemistry”, “ELISA”, “LC-MS/MS”, “proteomics”, “GC-MS” and “GC-MS/MS” (Figure 2). It amounted to 232, 133, 31, 23, 18, and 3, respectively, corresponding to approximate ratios of 73:44:10:8:6:1. This result suggests that the analytical methodology is likely to play a decisive role in the biomarker area of research and that very low numbers of articles may generate biased results.

### 4.5. Chronic Kidney Disease and Acute Kidney Injury

By using the search term “chronic kidney disease biomarker review” in PubMed, we found more than 1400 articles. The search “acute kidney disease [injury] biomarker review” resulted in some 900 articles. What can the present article contribute to this overwhelming information on biomarkers in kidney disease? Our literature search revealed a fraction of 8.1% for chronic kidney disease (CKD) and 10.7% for acute kidney injury (AKI) in combination with the term “biomarker” (Table 4). Because of the plethora of published articles on this topic, we will consider two recent review papers by Sapna, Kumar et al. on CKD [30] and by Yoon et al. on AKI [31], who searched PubMed and related databases.

#### 4.5.1. Chronic Kidney Disease

CKD typically shows no symptoms in its initial stages; significant kidney damage becomes apparent during stages 4 and 5, which typically leads to end-stage renal failure. According to Sapna, Kumar et al. [30], blood urea and serum creatinine values are mainly used to diagnose CKD, with serum creatinine showing low predictive ability. These authors expressed the opinion that new analytical methods (i.e., transcriptomics, genomics, epigenetics, proteomics, and metabolomics) have led to the discovery of new biomarkers in renal disorders. These potential biomarkers include:

➢ Neutrophil gelatinase-associated lipocalin (NGAL);
➢ Galectin-3 and kidney injury molecule-1 (KIM-1);
➢ Interleukin 18 (IL-18);
➢ Immunoglobulin G;
➢ Liver fatty acid-binding protein (L-FABP);
➢ ADMA and SDMA (see above and Table 6);
➢ miRNA biomarker, uromodulin, nephrin, epidermal growth factor (EGF), podocalyxin.

This group concluded that novel markers could replace traditional ones in the future, but evaluation of their effectiveness, sensitivity, and specificity is necessary [30].

#### 4.5.2. Acute Kidney Injury

AKI is a common condition that occurs in 5.0–7.5% of hospitalized patients and in 50–60% of critically ill patients. The current criteria for diagnosing AKI are a sharp decrease in glomerular filtration rate (GFR), as represented by an acute increase in serum creatinine levels or a decrease in urine output over a fixed period.

Yoon et al. [31] have provided a comprehensive review of biomarker-based AKI diagnosis and management and highlighted recent developments. According to these authors, an increasing number of studies have been conducted to standardize the definition and diagnosis over the past few decades. Along with serum creatinine level and urine output, more accurate novel biomarkers for predicting AKI are being applied for the early detection of renal dysfunction. A number of studies have demonstrated that new biomarkers are more sensitive in detecting AKI in certain populations than serum creatinine and urine output according to the recommendations from the Acute Disease Quality Initiative Consensus Conference.

Biomarkers for diagnosis of AKI include [31]:

➢ Serum cystatin C and proenkephalin A;
➢ Urinary tissue metalloproteinase-2 (TIMP-2) and insulin-like growth factor binding protein 7 (IGFBP7) are considered superior to KIM-1 and NGAL;
➢ Interleukin-18;
➢ Urine KIM-1 is a proven marker of AKI in adults;
➢ NGAL is a key polypeptide found in blood and urine at the time of AKI development after ischemic or toxicity-induced damage in the kidney;
➢ Alanine aminopeptidase; alkaline phosphatase; γ-glutamyl transpeptidase; hepcidin; *N*-acetyl-β-D-glucosaminidase.

Yoon et al. [31] stated in their review that there is a persistently unresolved need for earlier detection of patients with AKI. Biomarker-guided management may help to identify a high-risk group of patients progressing to severe AKI. However, these authors identified limitations such as biased data on certain studied populations and the absence of cutoff values for worldwide clinical use of biomarkers in the future.

Note that many of the potential “new” biomarkers mentioned above are also considered biomarkers in CKD. We also need to notice that these candidates are used in diseases of many other organs including the liver, heart and brain.

Table 9 shows the comparative results with respect to cystatin C (CysC), which is considered in about 46% of the articles as a biomarker in general and in 31% as a biomarker for kidney disease, both for CKD (11.6%) and AKI (10.8%). CysC is primarily considered a biomarker for the kidney (31.4%) rather than in the heart (8.1%), brain (3.8%) and liver (3.2%).

### 4.6. Cardiovascular Diseases

Cardiovascular diseases (CVDs) are related to the heart or blood vessels and include among others coronary artery disease (CAD), peripheral artery disease (PAD), and cardiomyopathy (CM). CVDs are often associated with other diseases such as diabetes and rheumatic diseases. Brain natriuretic peptide (BNP), NT-proBNP (60%) and troponin (37%) (Table 5), notably cardiac-specific troponins I and T, are extensively used as diagnostic and prognostic indicators for various CVDs such as congestive heart failure, myocarditis, myocardial infarct (MI) and acute coronary syndrome (ACS). About 10% of articles published in PubMed are related to CVD, but only 4.8% thereof refer to biomarkers in CVD, i.e., 4.4% for NT-proBNP and 7.7% for troponin, with troponin being the “earlier” CVD biomarker (Table 10). With respect to NT-proBNP and troponin, Table 5 and Table 10 indicate that the percentage fraction is dependent upon the reference parameter and can be misleading. Table 10 suggests that the kidney, brain and liver have been associated with CVD and biomarkers, but the majority of articles refer to the heart.

Cardiac troponin was introduced almost three decades ago and has been established since 2000 as the preferred biomarker for the diagnosis of myocardial infarction. In 2007, Melanson et al. [32] discussed cardiac troponin assays from commercial sources, which had been used at that time in clinical chemistry laboratories. The clinical application of cardiac troponin assays continues to progress in the analytical performance of commercially available cardiac troponin assays. Because of improved precision at low cardiac troponin concentrations and data establishing the prognostic relevance of quantitatively minor increases in cardiac troponin, the clinical decision limit has been pushed progressively lower. Research assays for cardiac troponin have reduced the limit of detection by 10- to 100-fold compared with current commercially available assays [33,34,35].

In their recent review, Yuan et al. [36] addressed several issues of ACS (see Table 11), including biomarkers. These authors stated that “the introduction of high-sensitivity cardiac troponins (hs-cTn) has revolutionized ACS diagnostic work-up, with earlier detection of myocardial injury at lower thresholds” and that “large real-world validation studies have confirmed that accelerated hs-cTn pathways achieve negative predictive values > 99% leading to reduced length of stay and no compromise in safety.”

In their review, Gao et al. [37] concluded that the clinical application of cTn testing continues to encounter numerous interference factors. These authors stated that LC-MS/MS presents a promising alternative. However, challenges persist regarding detection limits for clinical implementation of LC-MS/MS (LOQ values: 1.8 μg/L in LC-MS/MS vs. <5 ng/L hs-cTnI <5 ng/L). It is worth mentioning that LC-MS/MS is not suitable for the clinical use of other biomarkers such as NT-proBNP [38].

Takotsubo cardiomyopathy (TTS) is an acute, reversible cardiac condition marked by transient wall motion abnormalities of the left ventricle (see Table 11). Clinically, TTS and ACS overlap; there are no disease-specific diagnostic markers. A systematic review and meta-analysis by Kilaru et al. [39] reported that troponin showed limited diagnostic accuracy in differentiating TTS from ACS. BNP exhibited stronger discriminatory ability, whereas the combined use of BNP, troponin, and creatine kinase-myocardial band (CK-MB) enhanced diagnostic performance. The review by McKenzie and Bargout [40] came to similar results regarding non-invasive biomarkers to differentiate TTS from ACS on presentation: mild–moderate troponin elevation; marked BNP/NT-proBNP elevation; disproportionate rise in natriuretic peptides relative to troponin. Troponin and BNP provide non-specific corroborating data, and the serial biomarker profile is virtually indistinguishable from that of other cardiac diseases. Kilaru et al. [39], McKenzie and Bargout [40] and many other authors concluded that more validated innovative approaches are warranted on this topic. Omerovic and Redfors [41] emphasized in their review that the absence of randomized controlled trials for takotsubo syndrome underscores the urgent need for evidence-based therapeutic guidelines to refine care strategies.

### 4.7. Diabetes

Diabetes is characterized by sustained high blood glucose concentrations. The major types of diabetes are type 1 and type 2 (i.e., diabetes mellitus). Untreated diabetes can lead to various health complications. High concentrations of glucose and the glycated hemoglobin HbA_1C_ in blood serve as biomarkers for diabetes (due to insufficient production of insulin by the pancreas or insulin resistance) (see Table 12). Higher concentrations of HbA_1C_, which indicate persistent elevation of blood sugar, have been associated with cardiovascular disease, nephropathy, neuropathy, retinopathy and erectile dysfunction.

The use of HbA_1C_ for monitoring the degree of control of glucose metabolism in diabetic patients was proposed in 1976 by Koenig et al. [42]. Before diabetes treatment, the mean fasting blood sugar for all five patients was 343 mg per deciliter (19 mM), and HbA_1C_ concentration was 9.8%. During optimal diabetic control the blood sugar concentration was 84 mg per deciliter (4.7 mM), and the HbA_1C_ concentration was 5.8%. The authors found a correlation between the concentration of glucose and HbA_1C_ in the blood (r = 0.81, *p* < 0.01). This study showed that the HbA_1C_ concentration reflects the mean blood sugar concentration best over previous weeks to months. The authors suggested that periodic monitoring of HbA_1C_ concentration provides a useful way of documenting the degree of control of glucose metabolism in diabetic patients.

Are there specific biomarkers for diabetes type 2 (T2D) and diabetes type 1 (T1D), by which the two subtypes can be clinically discriminated?

The connecting peptide, or C-peptide, connects insulin’s A-chain to its B-chain in the proinsulin molecule. In their review, Jones and Hattersley [43] stated that C-peptide is produced in equal amounts to insulin and is the best measure of endogenous insulin secretion in patients with diabetes. Measurement of insulin secretion using C-peptide can be helpful in clinical practice: differences in insulin secretion are fundamental to the different treatment requirements of Type 1 and Type 2 diabetes. Briggs et al. [44] stated in their recent review that nonfasting, random C-peptide is suitable for diagnosis. Widely quoted cut-offs are <200 pM for T1D and >600 pM for T2D. These authors expected that in the future, C-peptide may have a role in diabetes sub-typing.

Using the search term “C-peptide” in combination with the search terms diabetes type 2 or diabetes type 1 revealed almost identical fraction values of about 12% (Table 12). Expectedly, a literature search in PubMed as conducted in the present article has no discriminating power.

### 4.8. Urogenital Tract

#### 4.8.1. Erectile Dysfunction

Erectile dysfunction (ED), also referred to as impotence, is a form of sexual dysfunction in men. ED affects up to 50% of men over 40 years. ED is attributed to physical risk factors and predictive factors, which are categorized as vascular, neurological, local penile, hormonal, and drug-induced. Besides aging cardiovascular disease (CVD), diabetes mellitus, high blood pressure, obesity, and hypogonadism are considered predictive factors.

The results of the search in PubMed found by using the indicated search terms relating to erectile dysfunction are presented in Table 13. Testosterone and nitric oxide achieved the highest rankings as biomarkers of erectile dysfunction. The very high ranking of prostaglandin E_1_ seems to be associated with ED mainly due to its use as a drug.

Earle and Stuckey [45] studied a cohort of 1455 men with a mean age of 54.9 years (range 16 to 82) referred for management of ED in their institute. Performed biochemical analyses included serum/plasma testosterone, prolactin, thyroxine, thyroid-stimulating hormone, random blood glucose, cholesterol, and iron. The prevalence of biochemical abnormality was 28% and 42% for cholesterol (*n* = 531). Abnormal findings were 5.7% in testosterone, 0.5% in prolactin, 0.13% in thyroid function tests, 12.8% in liver function tests 9.3% in glucose, 15% in cholesterol and 1.6% in ferritin. Testosterone and prolactin have a low yield, but specific therapy with a dopamine agonist for hyperprolactinemia and with testosterone for hypogonadism was effective. Glucose and lipids have a higher yield but specific therapy was not immediately effective for ED [45].

The overall expectation from a biomarker in the erectile dysfunction (ED) setting is to enhance the optimal management of a man with this disorder but without clinical atherosclerosis. Vlachopoulos and colleagues [46] investigated in their review a series of “robust” biomarkers for CVD within ED. In men with ED, total testosterone levels below 8 nM were found to be associated with increased risk of fatal major adverse cardiac events and subclinical organ damage. The measurement of prolactin may also be useful for CVD prediction within the ED population. The authors concluded that testosterone and biomarkers of generalized CVD may help to further quantify cardiovascular risk specifically in intermediate-risk ED patients. It is yet to be proven whether multiple biomarkers could be combined to improve performance for the prediction of CV risk in ED [46].

As a result of the discovery of nitric oxide (NO) and cyclic guanosine monophosphate (cGMP) as central mediators of penile smooth muscle relaxation, members of the L-arginine/NO pathway including NO metabolites and ADMA have attracted broad attention in the treatment of ED and its diagnosis [47].

Medication therapies in ED are based on the use of phosphodiesterase (PDE) 5 inhibitors such as sildenafil. PDE5 inhibitors inhibit the activity of the enzyme PDE5 that hydrolyzes and thus inactivates cGMP. Higher cGMP concentrations dilate blood vessels and facilitate increased blood flow into the penis. cGMP is produced by soluble guanylyl cyclase, whose activity is greatly enhanced by NO. This knowledge has presumably prompted some scientists to test the utility of the NO metabolites nitrite and nitrate as biomarkers, because endogenous NO itself evades analysis, and the inhibitor of NO biosynthesis ADMA as biomarkers for ED. In consideration of the very small part of vessels in the penis (compared to the whole-body vasculature) it is not surprising that the CVD biomarkers ADMA [48], nitrite and nitrate, even when measured in blood drawn from the penis [49], failed as biomarkers in ED [50]. The surprisingly high fraction of 10.9% of the search term “Erectile dysfunction biomarker nitric oxide” compared to ADMA, nitrite, nitrate, and cGMP (Table 13) presumably results from the general belief that the L-arginine/NO pathway plays a major role in ED. Yet, endogenous nitric oxide cannot be and has never been measured in human blood except after stimulation in situ [51]. The estimated half-life in human blood in vivo is less than 0.1 s [52].

This feature of NO resembles the instability of and inability to measure intact prostacyclin (PGI_2_) and thromboxane A_2_ (TxA_2_), two antagonistic eicosanoids [53]. ED has been associated with prostaglandins, almost entirely with prostaglandin E_1_ (PGE_1_), and with thromboxane, yet none of those have been considered as biomarkers of ED (Table 3).

PDE5 inhibitors are the first line of medical treatment for ED. Like NO, PGE_1_ is a vasodilator, but the mechanisms of their action differ (cGMP vs. cAMP). The drug alprostadil, i.e., PGE_1_ ethyl ester, has been investigated and used as a prodrug for PGE_1_ for topical application in ED [54]. Alprostadil is available in injectable, topical, and intra-urethral forms and offers an alternative treatment modality. Papadopoulos et al. [55] performed a systematic review and meta-analysis of 11 randomized controlled trials and 4 non-randomized studies (*n* = 5869). These authors emphasized the need for further high-quality, large-scale randomized controlled trials to confirm the improvement of ED symptoms by topical and intra-urethral alprostadil and to establish more definitive clinical guidelines for the use of alprostadil in the management of ED. It is also interesting to note the authors’ conclusion that the results of their analysis are limited by the variability in study designs and the low methodological quality of the included studies. We have often read such statements in reported meta-analysis publications.

Additional proposed biomarkers for ED include hs-C-reactive protein and more recently, endocan, adropin, and visfatin (Table 13).

The human corpus cavernosum consists mainly of smooth muscle and endothelial cells. The renin-angiotensin system and endothelium-derived vasoactive substances are involved in the control of arterial vascular tone in the penis. They include: angiotensin, endothelin-1 (ET-1), neuropeptide Y (NPY), oxytocin, arginine-vasopressin (AVP), vasoactive intestinal polypeptide (VIP), and differential peptide display (DPD). Despite some encouraging results, there is no convincing evidence for a biomarker associated with the above-mentioned peptides and enzymes.

#### 4.8.2. Prostate-Specific Antigen

Prostatic-specific antigen or p30 was discovered in the 1970s [56]. It is a 34-kD glycoprotein (P07288, UniProtKB (Swiss-Prot)). The term prostate-specific antigen (PSA) is a widely used misnomer: PSA is an antigen but is not specific to the prostate. PSA is present in large amounts in prostatic tissue and semen but also occurs in body fluids of men and women (e.g., amniotic fluid, breast milk, female serum and urine). PSA is secreted by the epithelial cells of the prostate gland in men and the paraurethral glands in women. PSA is not uniquely an indicator of prostate cancer but may also indicate prostate infection/inflammation, prostatitis, or benign prostatic hyperplasia (BPH). Prostate and prostate cancer, a life-threatening disease of men, have attracted the interest of clinicians and scientists from other disciplines including biology and analytical chemistry. The PSA reigns supreme as the prostate-specific biomarker (Table 14).

In the last two decades, many groups have performed mass proteomic studies on PSA. It is interesting to recall the results of two validation studies from a group by Semmes et al. [57], McLarran et al. [58] and colleagues who identified several challenges in the validation of PSA as a biomarker of prostate cancer. In the first study of this group [57], the authors reported that the SELDI-TOF MS technique itself was reproducible across 6 laboratories. The second study [58] revealed that the SELDI-TOF MS approach has no diagnostic value because of the inability of the validation study to identify men with prostate cancer. Discussed challenges include: (1) bias in serum specimens of earlier studies; (2) differences in study design; and, importantly, (3) analysis by SELDI-TOF MS of unprocessed/unfractionated serum samples. The authors suggested that all previous and forthcoming biomarkers should be subjected to equally extensive and rigorous validation.

The need for a detailed characterization and biochemical properties of PSA in the serum of prostate cancer patients and its normal form seen in seminal fluid is ever-increasing to explore the feasibility of whether or not the reliability of PSA serum assays can be enhanced. Bhanushali et al. [59] demonstrated how one should proceed in searching for biomarkers as exemplified for PSA. The authors described a detailed multiple-step analytical approach for the detection and verification of glycosylation patterns of PSA in human semen plasma, albeit not in a typical MS-based proteomic study. MALDI-TOF MS was used for the determination of the molecular mass, as well as the degree of purity of the seminal PSA protein sample. Bhanushali et al. [59] noted that the characterization and development of PSA assays require the highest possible purity to not only remove errors, if any, in the immune assays but also to enhance the reliability of the immunoassay.

The studies discussed above [57,58,59] clearly suggest that MS-based proteomic studies require considerable sample purification prior to MS analysis.

Ferraro et al. [60] addressed in their review the need to manage the gap between clinical and laboratory practice. The authors concluded that knowledge of the analytical issues and of their potential impact on the clinical decision-making of PSA has surely been lost. They emphasized that current clinical practice guidelines ignore the poor interchangeability of PSA results obtained from different assays. Knowledge of the selectivity of the antibodies employed in commercial PSA immunoassays can help to improve the reliability of the interpretation of the clinical results. Approximately 25% of the studies supported the evidence that nonselective PSA-based screening did more harm than benefit in the face of the considerable rate of false-positive results. In our opinion, these issues also apply to MS-based proteomic studies.

With respect to metabolomic studies in the area of PSA research, Lin et al. [61] stated that the so-far-reported metabolic signatures reflect aberrations across multiple metabolic pathways involved in prostate cancer. Reported data are based on non-targeted NMR in semen and GC/Q-TOF MS in urine samples. It seems that metabolomic studies in this area are the very beginning (Table 14); the metabolites (e.g., amino acids, fatty acids) measured in the blood and urine of the investigated patients with prostate carcinoma are common, and the suggested pathways are purely hypothetical and based on ROC-AUC values.

The authors of the present article hope that researchers are aware of the pivotal importance of their work for the life-threatening prostate cancer of men around the world. Even in the case of an “established” biomarker such as PSA, reliable quantitative analytical methods are indispensable, thereby also avoiding analytical pitfalls.

### 4.9. Muscular Dystrophy and Sclerosis

Muscular dystrophy (MD) is a genetically and clinically heterogeneous group of rare neuromuscular diseases. Muscular dystrophies are caused by mutations in genes responsible for the synthesis of muscle proteins. The muscle protein dystrophin is expressed in most muscle cells and is involved in muscle contraction and relaxation. Over 30 different disorders are classified as muscular dystrophies, with Duchenne muscular dystrophy (DMD) and Becker muscular dystrophy (BMD) being the most common. This is also reflected in the number of scientific articles (Table 15). The diagnosis of muscular dystrophy is based on the results of muscle biopsy, increased creatine phosphokinase (CpK3), electromyography, and genetic testing.

Sclerosis is the stiffening of a tissue or anatomical feature, usually caused by a replacement of the normal organ-specific tissue with connective tissue. Common medical conditions whose pathology involves sclerosis include multiple sclerosis (MS), amyotrophic lateral sclerosis (ALS), systemic sclerosis, and focal segmental glomerulosclerosis (FSGS) (Table 15). In 2020, nearly 3 million people were living with MS worldwide. There is no cure for any muscular dystrophy and sclerosis disorder thus far.

In a recent review/workshop [62], Marrie with colleagues and participants concluded that “considerable progress has been made in understanding genetic and environmental risk factors for MS, yet we still cannot prevent MS”. Regarding biomarkers the workshop participants recommended “identifying biomarkers relevant from initial immune dysregulation through to initial clinical presentation, linking biomarkers to long-term MS outcomes, developing cost-effective screening tools for MS and point-of-care testing, and developing prodromal criteria for MS that could be implemented clinically”.

In their review from 2014 [63], Comabella and Montalban stated that there is no specific biomarker for MS. They emphasized the need for validated MS biomarkers for clinical application. Thus far, diagnosis of MS is made by clinical examination, supported by MRI or CSF tests.

A proteomic study in CSF samples of three MS patients identified four proteins that were absent in the CSF of three non-MS subjects: CRTAC-IB (cartilage acidic protein), tetranectin (a plasminogen-binding protein), SPARC-like protein (a calcium-binding cell signaling glycoprotein), and autotaxin (ATX) (a phosphodiesterase) [64]. At that time (2004), it was unknown whether these proteins were related to the cause and pathogenesis of MS.

Since 2004, CRTAC-IB has not been mentioned in other articles. Tetranectin and SPARC-like protein are found in 242 and 50 PubMed articles, respectively, but only two of them are associated with MS. Autotaxin (ATX) is found in 1155 articles, of which 24 have been associated with MS and many other neurological and non-neurological diseases. ATX is considered to play pleiotropic roles in the nervous system [65]. In their review, Herr et al. [65] reported that currently (2020) there is one ATX inhibitor entering phase III clinical trials.

**Figure 3:**
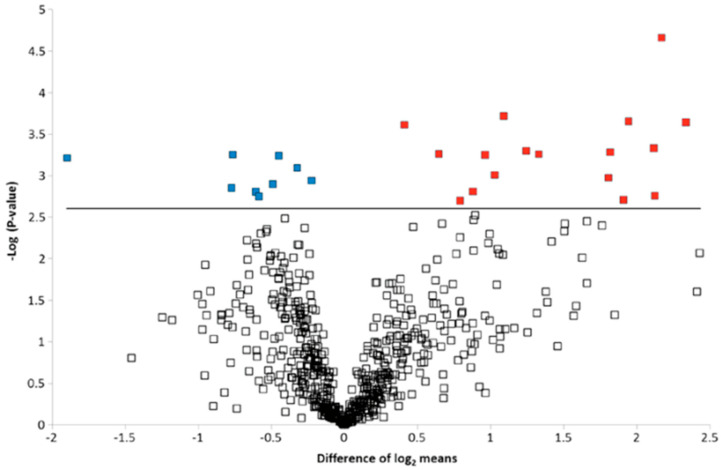
A typical Volcano plot from a proteomic study showing significantly dysregulated proteins [66]. The -log (*p*-value) is plotted against the difference in the means of the two groups (case and control). Points above the non-axial horizontal line are significantly differentially abundant proteins. Seventeen (17) proteins (red-colored squares) were found to be significantly (*p* < 0.002) more abundant in the case samples, while nine (9) proteins (blue-colored squares) were significantly less abundant in the case samples. Taken from Pavelek et al. [66].

A further proteomic study with LC-MS/MS investigated differences in the CSF proteome in 11 patients (cases) with relapsing-remitting MS (RR-MS) and in 15 healthy subjects (controls) [66]. The authors reported that 26 proteins (9 less abundant, 17 more abundant compared to the control) were significantly “dysregulated” in case samples compared to the controls (Figure 3). Three proteins were “uniquely” expressed in patients with RR-MS: (1) immunoglobulin (Ig) γ-1 chain C region; (2) Ig heavy chain V-III region BRO; and (3) Ig κ chain C region.

It is worth mentioning that proteins (1) and (2) have not been reported in other articles since 2016. In contrast, protein (3) is found in 681 articles, of which 4 have been associated with MS. In one of these articles (by Zhou et al., 1998), protein (3) had been detected in CSF from patients with non-neurological and other neurological diseases [67], suggesting that protein (3) is not “unique” for MS. It remains to be confirmed that protein (1) and protein (2) are indeed specific for MS.

MS is a chronic inflammatory autoimmune disease that primarily affects the white matter of the central nervous system. A recent study reported that 10% of people with MS have a common autoantibody signature in serum that is present before clinical presentation and that appears to be highly specific for MS [68]. This signature is a starting point for further immunological characterization of this MS patient subset and may be clinically useful as an antigen-specific biomarker for high-risk patients with clinically or radiologically isolated neuroinflammatory syndromes.

The study by Pavelek and colleagues on multiple sclerosis [66] is a typical systematic proteomic study. It delivers a large amount of data on proteins in a biological sample, typically CSF in MS, which may be useful in future studies. It shows that all 26 proteins are present in the CSF of both MS patients and controls, but they differ in their LC-MS/MS intensity. This gives us no information about their actual concentration and remains an uncertainty. Unfortunately, there are no subsequent studies by the same group in the same samples or by other groups to quantify the identified “dysregulated” proteins in clinical studies on a larger number of MS patients and non-MS patients/healthy controls by LC-MS/MS or immunological assays. This happens rather incidentally.

Stoevring et al. reported in 2006 a study on tetranectin (TN) in MS and other neurological diseases [69]. TN (see Ref. [64]) was determined in serum and CSF in 76 patients with MS and 47 controls and three other groups using a polyclonal sandwich ELISA [69]. All patients had lower concentrations of TN in the CSF than controls. To account for differences in blood–brain barrier permeability, the authors introduced and calculated the TN index. In definite MS and patients with a first attack of MS, the TN index was not significantly different from that of controls. The TN index and similar indices for other proteins are not used in proteomic studies to correct for blood–brain barrier permeability.

### 4.10. Pregnancy-Associated Diseases

In PubMed more than one million articles on pregnancy (gestation) are found, of which 22% (25%) are associated with gestational diseases (Table 16). Preeclampsia, peripartum cardiomyopathy and the HELLP (Hemolysis, Elevated Liver enzymes, and Low Platelet count) syndrome are multi-system disorders considered specific to pregnancy. Gestational diabetes (mellitus) accounts for a fraction of about 3%, and about 7% of the articles are associated with biomarker research.

#### 4.10.1. Preeclampsia

Preeclampsia is characterized by the new onset of high blood pressure and affects 2–8% of pregnancies worldwide. Peripartum cardiomyopathy (PPCM) is an idiopathic cardiomyopathy presenting with heart failure secondary to left ventricular ejection fraction (LVEF) below 45% towards the end of pregnancy or in the months following delivery. In PPCM, no cardiomyopathy or other cause of heart failure occurs prior to the last month of pregnancy. The HELLP syndrome is a complication of pregnancy. The HELLP syndrome occurs in about 0.7% of pregnancies and affects about 15% of women with eclampsia or severe preeclampsia. In the present work, we focus on PPCM, which has been the subject of intense scientific research in recent years also with respect to biomarkers.

#### 4.10.2. Peripartum Cardiomyopathy

PPCM is a life-threatening heart disease occurring in previously heart-healthy women. A common pathomechanism in PPCM involves the angiostatic 16 kDa-prolactin (16 kDa-PRL) fragment, which induces vascular damage and heart failure.

**Figure 4:**
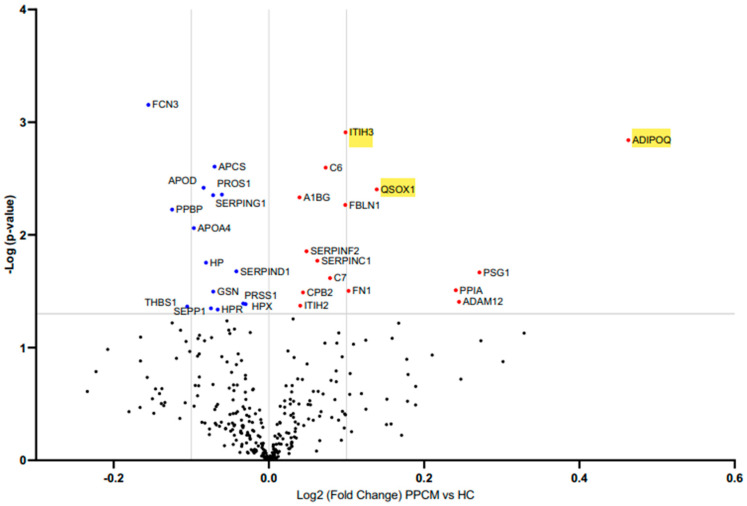
A typical Volcano plot from a proteomic study showing significantly dysregulated proteins in PPCM and PP-Ctrl [70]. Blue-colored symbols mean “down-regulated” and red-colored symbols mean “up-regulated” proteins. ADIPOQ, QSOX1, and ITIH3 are highlighted in yellow.

Kodogo and colleagues performed two proteomics studies in the serum of PPCM patients [70,71]. The first “untargeted” serum proteome profiling used data-independent acquisition–based label-free “quantitative” LC-MS/MS on 84 patients with PPCM, as compared with 29 postpartum healthy controls (PP-Ctrl). The authors identified 15 differentially “up-regulated” and 14 “down-regulated” proteins in patients with PPCM compared with HCs (Figure 4). The combination of adiponectin (ADIPOQ), quiescin sulfhydryl oxidase 1 (QSOX1), inter-α-trypsin inhibitor heavy chain (ITIH3A), and NT-proBNP was stated to have the best diagnostic precision to distinguish patients with PPCM from PP-Ctrl. Immune response proteins, inflammation, fibrosis, angiogenesis, apoptosis, and coagulation were found to be predominant in patients with PPCM compared with PP-Ctrl.

In the second “targeted” proteomic study by Kodogo et al. [71], 82 PPCM patients of different ethnic groups, but no control group were included. It was not explicitly stated whether the 82 PPCM patients belonged to the same cohort of 84 PPCM patients of the first study [70]. Serum samples were processed, digested and analyzed by using data-independent acquisition-based label-free “quantitative” LC-MS/MS as reported in the first study [70]. The authors did not find ethnic differences in the expression of ADIPOQ, QSOX1 and ITIH3A, which had been observed in the first study [70]. The authors did not report concentrations for ADIPOQ, QSOX1 and ITIH3, but relative abundances for these proteins.

Ricke-Hoch and colleagues [72] performed an extensive clinical study on healthy age-matched postpartum women (PP-Ctrl, *n* = 53) and on postpartum PPCM patients at baseline (BL, *n* = 64) and at 6-month follow-up (*n* = 23). Laboratory workup was performed as part of routine analysis by hospital laboratories for NT-proBNP, high-sensitivity CRP, troponin T, and creatine kinase. Plasminogen activator inhibitor-1 (PAI-1), IL-6, IL-1β and other biochemical parameters were measured in plasma by commercially available assays.

In healthy postpartum women, the plasma levels of PAI-1 were within the normal range, significantly higher in postpartum PPCM patients at baseline, and then decreased at the 6-month follow-up (Table 17). Note the great inter-individual variability of the measured biomarkers even in the control group. The authors concluded that measuring circulating PAI-1 and miR-146a, together with an uPAR/NF-κB-activity assay could be developed into a specific diagnostic marker assay for PPCM.

A recent literature search was performed by Pretorius and colleagues [73] in PubMed, Scopus, and Web of Science for studies relating to PPCM and published between January 2000 and August 2025. Conventional biomarkers such as BNP and NT-proBNP lack specificity in PPCM. The authors stated that recent advances led to novel markers in angiogenic pathways:

➢ Cathepsin D;
➢ miR-146a;
➢ The sFlt-1/PlGF ratio.

Pretorius et al. [73] identified diverse emerging biomarker candidates, which reflect diverse mechanisms:

➢ VEGF (vascular endothelial growth factor);
➢ PlGF (placenta growth factor);
➢ ADIPOQ;
➢ QSOX1.

These authors concluded that multi-marker strategies are essential to address PPCM heterogeneity and improve diagnostic accuracy [73].

#### 4.10.3. Gestational Diabetes Mellitus

Li and colleagues [74] performed a metabolomic study by GC×GC–TOF/MS on 48 diabetes mellitus patients (not further specified) and on 31 healthy subjects as a control. Five potential biomarkers including glucose, 2-hydroxyisobutyric acid, linoleic acid, palmitic acid and phosphate were identified. Elevated free fatty acids were found to be essential pathophysiological factors in diabetes mellitus. These authors concluded that these potential biomarkers in plasma, e.g., palmitic acid, linoleic acid and 2-hydroxybutyric acid might be helpful in the diagnosis or further study of diabetes mellitus.

Dudzik and colleagues [75] performed a metabolomic study on plasma and urine samples from 20 women with gestational diabetes mellitus (GDM) and from 20 pregnant women with normal glucose tolerance in the second trimester of pregnancy. The authors used three previously described analytical techniques, i.e., LC-QTOF/MS [76], GC-Q/MS [77] and CE-TOF/MS [78], and found considerably lower (by up to −77%) and higher (up to +75%) concentrations of more than 500 metabolites. GC-Q/MS analysis revealed differences in the range of -54% to +75%. The highest differences were found in plasma for 2-hydroxybutyric acid by +68% and for 3-hydroxybutyric acid by +75% in the GDM patients. No changes were reported for stearic acid in the study.

In a subsequent study on GDM, Dudzik and colleagues performed a metabolomic study and measured among others 2-hydroxybutyric acid and 3-hydroxybutyric acid as trimethylsilyl ester derivatives by GC-Q/MS in the electron ionization (EI) mode using stearic acid methyl ester as an internal standard for these and all other metabolites [79]. The authors did not report absolute concentrations of metabolites but their relative changes.

In the second trimester of gestation, the plasma concentrations of 2-hydroxybutyrate were 51% higher, and those of 3-hydroxybutyrate were 81% higher compared to control. Three months postpartum, the plasma concentrations of 2-hydroxybutyrate were 37% higher, and those of 3-hydroxybutyrate were 128% higher in the GDM women compared to healthy controls (C). In the second trimester of pregnancy of GDM, in women who were diagnosed with T2DM within two years after delivery (i.e., GDMT2DM) and in GDM women with normoglycemia (i.e., GDM-C), the plasma concentrations of 2-hydroxybutyrate were 94% higher, and those of 3-hydroxybutyrate were 249% higher compared to GDM-C. Three months postpartum, the plasma concentrations of 2-hydroxybutyrate were 161% higher, and those of 3-hydroxybutyrate were 1511% higher in GDMT2DM compared to GDM-C. These values suggest a much higher elevation of the 3-hydroxybutyrate levels compared to 2-hydroxybutyrate.

**Figure 5:**
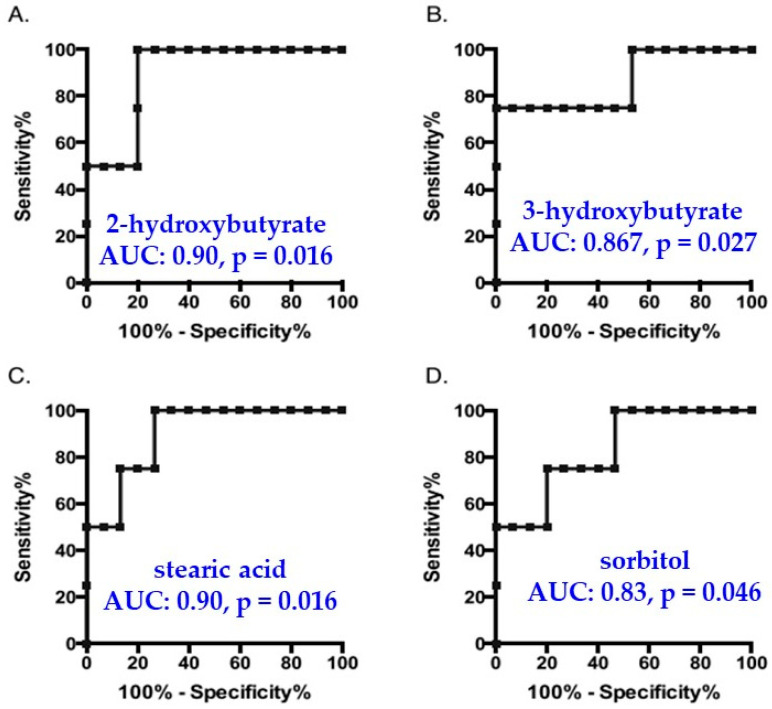
ROC curves of selected metabolites in the second trimester of gestation, including GDM women who were normoglycemic or those with confirmed postpartum diabetes mellitus. With permission of Dudzik et al. 2017 [79].

Figure 5 shows the receiver-operating characteristic (ROC) curve analysis and area under the curve (AUC) values for 2-hydroxybutyric acid, 3-hydroxybutyric acid, stearic acid and sorbitol of selected metabolites in the second trimester of gestation, including GDM women that were normoglycemic (GDM-C) and those with confirmed postpartum diabetes mellitus (GDMT2DM). It is interesting to note that the AUC values of 2-hydroxybutyric acid, 3-hydroxybutyric acid, stearic acid and sorbitol were of the same order of magnitude.

As mentioned above, stearic acid did not belong to the analytes that exhibited the most significant changes with gestational diabetes in the first study [75]. In the second study [79], the plasma concentration of stearic acid was measured to be +49% higher in GDMT2DM compared to GDM-C three months postpartum, i.e., much lower compared to 2-hydroxybutyric acid and 3-hydroxybutyric acid. Dudzik and colleagues [79] concluded in their article that “2-hydroxybutyrate and 3-hydroxybutyrate may be considered as future prognostic biomarkers to predict the onset of diabetic complications in women with gestational diabetes after delivery”.

Targeted metabolomic studies were performed by other researchers. Xu et al. [80] detected 46 differential metabolites (24 “upregulated” and 22 “downregulated”) between GDM and normal pregnancy. The authors reported AUC values above 0.75 for 2-hydroxybutyric acid, itaconic acid, *O*-acetylcarnitine, glutathione disulfide, *p*-cresol sulfate, 2-furoic acid, L-asparagine, d-biotin, choline and homovanillic acid. Kong et al. [81] found elevated concentrations of pyroglutamic acid, γ-aminobutyric acid, glutamic acid, oleic acid, and palmitic acid. It is notable that pyroglutamic acid can be formed artefactually from glutamate and γ-glutamyl peptides such as glutathione [82], including by derivatization with BSTFA [82,83]. Glutamate/glutamine is among the most abundant amino acids in human plasma.

At this point we should point out that the previous sections revealed one of the big challenges in the area of biomarker research, which is the receiver-operating characteristic (ROC) curve analysis with the area under the curve (AUC) being its single value. ROC analysis enjoys a great deal of popularity in high-throughput analytical technologies including proteomics and metabolomics. ROC and AUC are considered the most objective methods for evaluating and comparing classification performances and are recommended for the evaluation of binary classifiers [84]. Several authors have pointed out caveats and pitfalls of the ROC-AUC approach and criticized certain applications of the ROC curve analysis and AUC as measurements for assessing binary classifications when they do not capture the information relevant to the application [85,86,87,88,89]. According to Berrar and Flach [86], a key point is that the AUC value cannot directly measure a clinical improvement or loss, which is the primary outcome in clinical settings. Chicco and Jurman [89] criticized that the ROC-AUC approach says nothing about precision and negative predictive value.

### 4.11. Post-Translational Modifications—Dimethylation of Arginine to ADMA and SDMA

The so-called post-translational modifications (PTMs) of amino acid residues in proteins may have far-reaching consequences for health and disease [90]. The first scientific publications appeared as early as the 1940s and evolved drastically over the last two decades, indicating strong interest in PTMs by researchers from many different disciplines (Table 18; see also Table 8). A search in PubMed using the term “posttranslational modifications proteins” resulted in 106,391 articles in the period from 1946 until 2025. PTMs occur virtually in all kinds of amino acid residues in numerous proteins and are catalyzed by specific enzymes. PTMs of proteins include acetylation, methylation and glycation in addition to the most widely occurring PTMs phosphorylation and ubiquitination (Table 8). Glycation of the *N*-terminal valine of hemoglobin generates HbA_1c_, one of the earliest indices of diabetic control [91].

**Figure 6:**
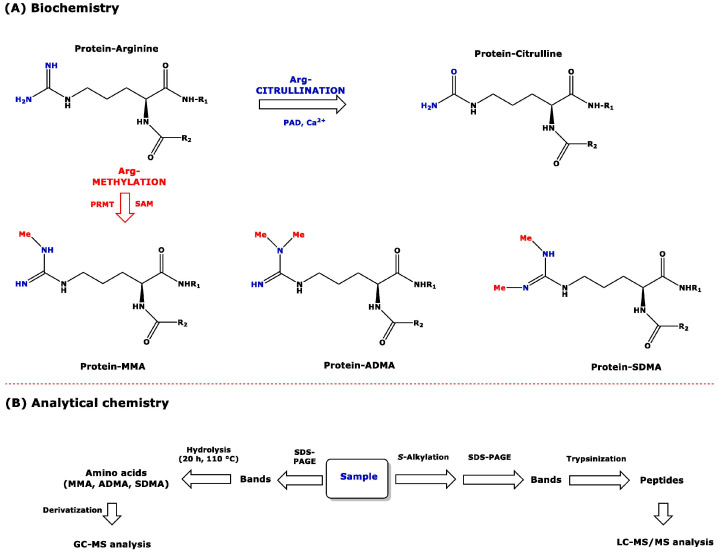
(**A**) Simplified scheme of two major post-translational modifications of proteinic L-arginine, i.e., PRMT-catalyzed monomethylation of L-arginine to monomethylarginine (MMA), asymmetric dimethylarginine (ADMA) and symmetric dimethylarginine (SDMA) proteins, and PAD-catalyzed citrullination of L-arginine in proteins. (**B**) Schematic of the analytical workflow for the identification of (a) symmetrically methylated L-arginine peptides by LC-MS/MS and for the quantitative determination of amino acids by GC-MS. For LC-MS/MS analysis, proteins were first *S*-alkylated by acrylamide and then separated by SDS-PAGE; bands were isolated, proteins were trypsinized and the obtained peptides were separated by liquid chromatography and finally identified by tandem mass spectrometry (MS/MS). For GC-MS analysis, proteins were separated by SDS-PAGE, bands were isolated, amino acids were released from proteins by HCl-catalyzed hydrolysis and derivatized by 2 M HCl/methanol followed by pentafluoropropionic anhydride. Me, methyl; PAD, protein-arginine deiminase; PRMT, protein-arginine methyltransferase; SAM, *S*-adenosyl-methionine. With permission of Bollenbach et al. 2020 [100].

Arginine residues undergo abundant citrullination and mono- and dimethylation (Figure 6) as it resulted from measuring ADMA and its hydrolysis product dimethylamine (DMA), and of SDMA in human urine [92] (see also Section 4.1). PTMs have been associated with human disease (fraction, 34.6%), and a fraction of 2.9% seems to be associated with biomarker research (Table 18). Citrullination is considered to play a particular role in rheumatoid disease [93]. The significance of *N*^G^-methylated proteins in health and disease is still unexplored. The mono- and di-methylated proteins can be expected to have numerous functions in the human body. Thus far, most studies have focused on the identification of Arg-methylated proteins and the sites of their methylation. It is known for several decades that proteolysis of *N*^G^-monomethylated proteins and dimethylated proteins releases free MMA, SDMA and ADMA, which are endogenous inhibitors of the activity of NOS isozymes including neuronal NOS [6,7].

Zinellu and colleagues [94] used capillary electrophoresis (CE) with fluorescence derivatization and measured MMA-, ADMA- and SDMA-proteins, i.e., pMMA, pADMA and pSDMA, respectively, in the whole blood of 134 elderly healthy subjects. The mean concentrations (nmol/mg protein) were determined to be 4.31 for MMA, 4.11 for pADMA and 1.66 for SDMA, with no differences between males and females [94].

Wang and colleagues recently reviewed the functional dynamics of Arg-methylation from a biochemical perspective [95]. These authors analyzed 15 published proteomic data sets and found 12,742 MMA sites across 4832 proteins and 2658 DMA sites across 999 proteins. pADMA are believed to be implicated in muscle atrophy and kidney diseases, whereas pSDMA are considered to be associated with prostate cancer [95]. Table 18 shows that cancer is by far one of the most widely investigated diseases in the context of MS-based proteomics of PTMs. Arg-methylated proteins are thought to contribute to breast cancer development. However, these are rather expectations, which are likely to originate from the general belief that methylation is generally associated with cancer (Table 18). It is notable that the titles of many articles on proteomic studies in this research area are suggestive and misleading.

Protein-arginine methyl transferases (PRMT) and the methylation cofactor *S*-adenosyl-methionine (SAM) (Figure 6), the common cofactor of all methyl transferases, are omnipresent. It seems that Arg-methylated proteins are ubiquitous too, but their quantitative analysis in biological samples from clinical studies including both patients and healthy controls is very fragmentary.

We wish to mention here three examples. Lim and colleagues [96] performed a proteome-wide identification of Arg-methylation in proteins of colorectal cancer tissues from 10 patients, yet not in cancer-free tissue samples. Tan and colleagues [97] performed a proteome-wide identification of pADMA in pancreatic tissue of 36 patients with chronic pancreatitis, yet not in healthy controls. Wei and colleagues [98] performed proteomic studies in cell experiments. It is notable that in these studies no quantitative analyses of pMMA and pADMA had been performed.

There is no convincing evidence so far of the involvement of Arg-methylated proteins in human disease, in contrast to the free ADMA and SDMA as discussed in Section 4.1. The quantitative relationship between Arg-methylated proteins and their products of proteolysis, the LMM metabolites MMA, ADMA and SDMA, remains to be explored. Are possibly pADMA and pSDMA rather than the free soluble ADMA (fADMA) and free soluble SDMA (fSDMA) responsible for their involvement in human disease, and can this explain the ADMA-SDMA paradox [7]?

In a small study on healthy elderly human subjects (*n* = 27), the mean concentrations in serum samples were determined to be 0.493 µM for fADMA and 0.113 µM for pADMA (from HCl-catalyzed hydrolysis) [99]. This observation indicates that in serum the concentration of pADMA is around 4 times lower than that of fADMA. The study showed that the serum concentrations of fADMA and pADMA did not differ between non-infected and *Helicobacter pylori*-infected subjects.

Human serum albumin and human hemoglobin do not contain pADMA. Yet, human red blood cells are rich in large (>50 kDa) pADMA. The concentration of pADMA in lyzed erythrocytes from healthy humans is estimated to be up to 20 µM, i.e., up to 50 times higher than that of fADMA. An LC-MS/MS proteomic study in human hemolysate has been performed after removal of the largest portion of erythrocytic hemoglobin by means of commercially available HemoVoidTM cartridges (Figure 6) [100]. Several membranes and cytosolic erythrocytic *N*^G^-dimethylated proteins were identified, including spectrin-α (280 kDa), spectrin-β (247 kDa) and protein 4.1 (80 kDa). Being responsible for the stability of the erythrocyte membrane, the newly identified targets for *N*^G^-dimethylation in human erythrocytes were proposed to be involved in erythrocytic diseases such as hereditary spherocytosis and sickle cell anemia [100]. So far, it is unknown whether Arg-dimethylation of erythrocytic membrane proteins has a positive or negative effect on the stability of the erythrocyte membrane. In theory, human erythrocytes could serve as a pool for fADMA in the circulation [100].

### 4.12. Challenges and Limitations of Discovering New Clinical Biomarkers

Physiological LMM and HMM substances belong to various chemical classes. Their number in the human body is not yet known. Thus far, proteomic analyses by MS-based and non-MS-based techniques have identified around 20,000 proteins of the human proteome [101]. According to the Human Metabolome Database (HMDB; (https://hmdb.ca/)), the number of identified metabolites amounted to around 110,000 in the year 2018 and already to 220,000 in 2022 [102,103] (see Table 19). In living organisms such as the human body, physiological substances are inter-related and the majority of them have a typical life: synthesis, metabolism and elimination in specific cells of different organs, which are also inter-related and inter-connected by the circulating blood. The amounts/concentrations of physiological and non-physiological substances (e.g., drugs) in human biological fluids and tissues change continuously and persistently in dependence on various endogenous and exogenous factors such as age, gender, daytime, lifestyle, and intake of food and water. The concentrations of physiological substances in certain compartments such as blood and urine lie within greatly overlapping ranges.

In clinical medicine, more or less arbitrarily predefined reference values and intervals for biochemical and functional parameters are commonly consulted in classifying qualitatively (e.g., healthy, diseased; normal or elevated blood pressure) and quantitatively (e.g., severely diseased) the state of health. In principle, every physiological substance can serve as an indicator and even as a measure of a certain disease, i.e., as a biomarker. From an analytical point of view, this vagueness is rather a curse than a blessing. In practice, only a handful of substances deserve the name disease-specific biomarker. Prominent examples are diseases due to inborn errors, which result in highly elevated concentrations in the blood and/or excretion in the urine of certain amino acids and metabolites such as phenylalanine in phenylketonuria and citrulline in citrullinemia. In diabetes, glucose metabolism is impaired leading to elevated concentrations of glucose and HbA_1C_ in the blood. Identification of disease-specific biomarkers can save lives and help control the respective disease by taking proper measures including diet and drugs.

Discovering and establishing new biomarkers is highly challenging, time-consuming and costly. In the preceding sections, we addressed explicitly some widely used clinical biomarkers such as the prostate-specific antigen PSA. Yet, its utility as a biomarker for prostate cancer has many grave limitations, which are consistently discussed in the literature. Regardless, PSA is routinely measured in blood, semen or prostate tissue around the world for several decades by order of urologists, and its levels are used complementarily to other clinical measures. The use of PSA as a biomarker is fraught with several difficulties including its analytical measurement, the nature of the human being per se, or other apparently hardly conceivable reasons. As mentioned at the beginning of the article, this especially applies to the so-called biomarkers of oxidative stress, of which at least 71 have been reported in the literature [5], including the most widely used malondialdehyde.

It is notable that oxidative stress biomarkers have been reported to occur in practically all examined human diseases and to indicate elevated oxidative stress, yet without previous validation and an agreed definition of reference values and intervals for the majority, if not even for all used oxidative biomarkers. The authors of the present article are not aware that any of the dozen biomarkers of oxidative stress, especially including malondialdehyde and 8-*iso*-prostaglandin F_2α_, have been clinically validated by scientifically sound approaches. We suggest calling this kind of biomarker an experimental biomarker.

Recent studies provided strong indications that in many human diseases, such as in peri/postpartum cardiomyopathy (PPMC) (e.g., [70,71,72]) and gestational diabetes (e.g., [74,75,79,80,81]), there exists no single disease-specific biomarker. Rather, it seems to be advisable to consider a bundle of biomarkers (see Section 4.10).

Analogously, substances such as ADMA and SDMA, which are found in elevated concentrations in various diseases but are not specific to particular diseases and lack a specific biological mechanism, also deserve to be named experimental biomarkers.

### 4.13. Analytical Chemistry in Clinical Biomarker Research—Prospects and Requirements

Table 19 provides an overview of the main analytical methods used in the research of human biomarkers. They include chromatographic (HPLC, GC-MS, LC-MS/MS), spectroscopic (NMR), electrophoretic (CE) and immunological (RIA, ELISA, immunohistochemistry) methods.

Advances in hyphenated MS-based techniques and computer technology in the last 2 to 3 decades, in part driven by commercial companies, have “revolutionized” and extended exponentially the areas of application of MS in experimental and clinical research. Commonly, “special” software is provided by manufacturers for proteomic and metabolomic studies to acquire, analyze, evaluate, and even profile the mass spectrometric signal, largely in an automated fashion. Little or no freedom is given to researchers to intervene.

The complexity of biological samples, in part very low concentrations of certain biomarkers in the presence of high concentrations of their precursors and of other analytes, e.g., differences of up to 10^10^ between some cytokines and albumin [104], and LMM metabolites, such as mM-concentrations of glucose in blood and of creatinine in urine, and many other factors (e.g., instability and artefactual formation during sample storage) represent formidable analytical challenges, even for the MS technology, the *Gold Standard* in chemical analysis. Unobjectionable qualitative and quantitative analysis is indispensable in general and in the biomarker research area in particular [5,24].

**Figure 7:**
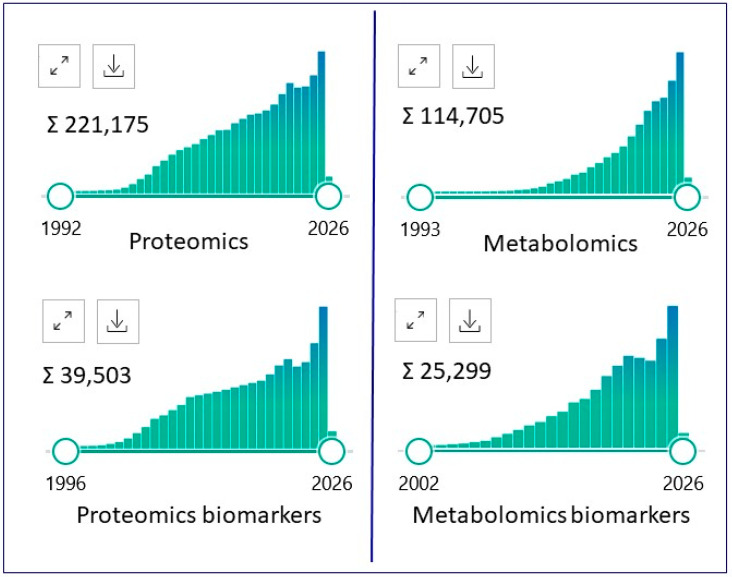
Original graphs showing the time course of publications found in PubMed by using the search terms proteomics or metabolomics alone and in combination with the term biomarkers since 1992. Retrieved on 25 January 2026. Insets indicate the total number of articles (Σ).

Our present literature search indicates that proteomics (i.e., large-scale study of proteins) and metabolomics (i.e., large-scale study of LMM metabolites) provide researchers with boundless possibilities to analyze hundreds of biological samples within a short time (high-throughput analyses) for hundreds of proteins and LMM metabolites, respectively. About every fifth proteomic and metabolomic article seems to be associated with biomarkers (Figure 7). Yet, do these modern MS-based analytical techniques keep their promises? Below we address these issues in some detail.

Metabolomics. The proportion of human metabolites that can be identified via untargeted MS-based metabolomics techniques is typically below 2% of detected MS peaks, and less than 10% of known human metabolites have authentic, experimentally collected NMR, GC-MS, or MS/MS spectra. These data suggest that existing chemical compound data sets and existing experimental spectral data sets for metabolomics are far too inadequate for comprehensive metabolite identification or quantification [102] (Table 20). In the HMDB, it is believed that in silico metabolomics, i.e., the generation of data not by experiments but by computers will overcome the “crisis” of metabolomics and will allow users to more accurately identify or annotate metabolites [103]. We do not think that this is a good solution: the generation of spectra by NMR, MS, and MS/MS by using fully characterized pure LMM reference substances is indispensable for the generation of reliable scientific databases.

Yet, the impact of artificial intelligence (AI) is exponential and unstoppable. Within less than 6 months, the number of articles found by using the search term “human biomarker artificial intelligence” increased from 16,909 (Table 19) to 19,501, i.e., by around 15%. This evolution does not keep pace with rational scientific and clinical needs [3].

*Proteomics*. Like metabolomics, proteomic analysis is highly amenable to automation and large data sets are created that are processed by software algorithms. Filter parameters are used (not without arbitrariness) to reduce the number of “false” hits. Protein identification usually relies on the incomplete coverage of peptide sequences output from MS analysis and returns information about matching or similar proteins through alignment algorithms with proteins from known databases to predict what proteins are in the sample with a degree of certainty (Table 20). Scientists have expressed the need for awareness that proteomic studies should adhere to the criteria of analytical chemistry, e.g., sufficient data quality, sanity check, and validation [24,105,106,107]. It is worthy of mention that proteins undergo numerous and in part abundant post-translational modifications, such as citrullination and *N*^G^-dimethylation of Arg residues to isomeric asymmetric and symmetric arginine residues (see Section 4.11). Yet, the concentration of proteins modified by PTMs is much smaller than the native non-modified proteins, e.g., the molar ratio accounts for 1:10^6^ for 3-nitrotyrosine to tyrosine in human serum albumin [24]. The expected effects of PTMs on MS-based proteomic studies in humans have not been investigated properly so far.

*Quantitative analysis in proteomics and metabolomics*. Quantitative determination of the concentration of candidate biomarkers—including ensuring metrological traceability—is of decisive importance in their discovery, validation and application in clinical settings. By nature, quantitative analysis of low-abundance proteins, to which this category belongs the vast majority of biomarker proteins, is extraordinarily difficult, mainly because of the inherent lack of stable-isotope labeled proteins for use as internal standards in MS-based proteomic studies. The value of proposed alternatives is limited in our opinion and can provide only first estimates. This kind of limitation also applies to metabolomic studies. In fact, targeted proteomic and targeted metabolomic studies using LC-MS/MS cannot provide concentrations as can be provided by validated quantitative LC-MS/MS methods that use matched isotopologues for each analyte. In our opinion, it is not correct to characterize as quantitative LC-MS/MS methods that are currently used in typical proteomic and metabolomic studies by using LC-MS/MS or GC-MS, respectively.

It is worthy of mention that in the vast majority of performed proteomic and metabolomic studies, no quantitative analyses are subsequently performed on the same study samples (see for instance [75,79,80,81]). Thus, proteins and metabolites initially detected, for instance by Volcano plots (Figure 4 and [70]), as “up-regulated” and “down-regulated” in the samples and as potential candidates as disease biomarkers remain in principle “unvalidated”. It is interesting to note that the Volcano plot was originally reported and applied in genetics [108]. It was adopted and is increasingly used in proteomics and metabolomics since about 2010. This also applies to the ROC-AUC approach, which experiences its renaissance notably in metabolomics.

The Volcano plot helps quickly pinpoint proteins and metabolites with significant changes in expression and concentrations between diseased and healthy subjects. In addition, the ROC curve analysis approach is also used and can provide some “quantitative” statistical results by using AUC values. As discussed above (see Section 4.10.3), the ROC-AUC approach is associated with caveats and pitfalls. More importantly, cutoff values beyond statistical issues have not been defined so far, neither for the Volcano plot nor for the ROC plot, for instance for AUC values. Which AUC values of candidate biomarkers are high enough for their consideration in forthcoming studies? We have recently suggested using for this purpose data from substances such as creatinine, which are known to differ in diseased and healthy subjects [109], analogous to the use of creatinine excretion for the standardization of metabolites measured in urine collected by spontaneous micturition.

In the study by Kodogo et al. on PPCM [70], the Volcano plot showed 15 “up-regulated” and 14 “down-regulated” and ROC analysis revealed AUC values of 0.59–0.70 for single proteins, 0.72 to 0.86 for a combination of two proteins, and 0.898 for the combination of three proteins. Interestingly, the highest AUC value of 0.808 was observed for NT-proBNP, a protein that the Volcano plot did not classify as dysregulated [70]. These observations reveal one major dilemma of proteomics.

Another important factor, a biological factor that is per se independent of chemical analysis, is the inter-individual variability of endogenous substances including the biomarkers in health and disease. For instance, the endogenous LMM substance ADMA has a relatively low inter-variability of 12% to 17% [110,111], while other (candidate) biomarkers, such as PAI-1, CRP, hsTNT and NT-proBNP (see Table 17), have much higher ranges. Clinical studies performed over the last three decades by different groups using different kinds of analytical methods on humans suffering from different diseases have consistently revealed that the differences in ADMA plasma concentrations of healthy and ill individuals, notably in cardiovascular diseases, and at various stages of pharmacotherapy (10%), are also very low, independent of the analytical method [111]. Low inter-individual variability has been proposed to characterize an “ideal” biomarker, at least in the area of oxidative stress [5]. Reliable quantitative analysis by various techniques including HPLC, ELISA, GC-MS/MS and LC-MS/MS is likely to have contributed to the establishment of ADMA as a biomarker [112]. Nevertheless, there is no specific disease to which ADMA could be assigned (see Section 4.1). Given the very low inhibitory potency of ADMA towards eNOS (around 12 µM) compared to its low plasma concentration (around 0.4 to 0.5 µM) there is still no convincing biochemical evidence for the justification of ADMA as a biomarker. ADMA was characterized more than 20 years ago as *Übermarker* (German word; “over marker” or “super marker” word-for-word translation), a biochemical factor mediating the adverse vascular effects of many other risk factors and markers [113]. Today, we can extend the term *Übermarker* to SDMA for which no biochemical rationale is available so far.

Sample preparation is of enormous importance for reliable qualitative and quantitative analysis of LMM and HMM substances. Chemical and immunoaffinity chromatography is the most efficient and elegant technique for specific extraction and enrichment but is strongly limited for selected analytes notably for proteins. Affinity chromatography has found application in MS-based proteomics [114,115,116,117]. Lu and colleagues [118] have recently reported the “pan-specific” enrichment of methylarginine-containing peptides based on the Tudor domain of SMN, a naturally occurring methylarginine reader protein, for comprehensive proteomic profiling of cellular arginine methylation. Use of affinity chromatography in proteomics is expected to increase the specificity of proteomic research on human-disease biomarkers [118].

An important analytical issue in omic studies is the involvement of quality control systems. In some “targeted” and “untargeted” metabolomic studies, quality control systems have been implemented; see for instance Gika et al. 2016 [119] and Märtens et al. [120], and are currently used. Yet, in the vast majority of metabolomic studies quality control is usually limited to precision (e.g., [74,75,76,77,78,79]). There is a need to include in quality control systems the measurement of accuracy as has been proposed by us for the analysis of amino acids [121].

### 4.14. Clinical Chemistry in Routine Diagnostics

Laboratory medicine, and clinical chemistry in particular, comprises the analysis of measurable biological parameters to assess pathological or physiological alterations. Results derived from these investigations play a pivotal role in diagnosis, disease monitoring, prognostic assessment, therapeutic decision-making, and prevention.

Clinical chemistry laboratories rely predominantly on fully automated, high-throughput analytical platforms integrating pre-analytics, sample transport, reagent handling, calibration stability, internal quality control measurements, and bidirectional laboratory information system (LIS) connectivity.

Widely used core technologies include spectrometric assays for small molecules and enzymes (e.g., glucose, creatinine, urea, bilirubin, aminotransferase, creatine kinase), ion-selective electrodes (for electrolytes), particle counting, and immunoassays—such as immunoturbidimetry, chemiluminescent or electrochemiluminescent methods—for protein biomarkers including inflammatory markers such as C-reactive protein (CRP), cardiac markers (e.g., troponins), and hormones such as TSH (thyroid-stimulating hormone).

Automated core-laboratory platforms supplied by major in vitro diagnostics manufacturers operate as integrated, validated, high-throughput systems using dedicated reagent kits, calibrators, and quality control materials. In the United States, in vitro diagnostic devices are regulated by the U.S. Food and Drug Administration (FDA) [122]. In the European Union, conformity assessment follows Regulation (EU) 2017/746 on in vitro diagnostic medical devices (IVDR) repealing Directive 98/79/EC [123].

These user-friendly and powerful high-throughput approaches are well established but can have their limitations in analytical specificity and selectivity. Mass spectrometry, notably LC-MS/MS, a highly specific and selective technology, is primarily used in specialized clinical laboratories, for instance for newborn screening, therapeutic drug monitoring (TDM), in endocrinology or toxicology [124]. For routine diagnostics, commercial MS kits can help simplify the application of this complex technology. Kit manufacturers provide either complete kits or individual components, such as calibration materials, which must comply with the IVDR. The manufacturer is required to provide a conformity assessment demonstrating that the products meet the safety and performance requirements of Annex I of the IVDR. Additionally, manufacturers must operate under a quality management system in accordance with DIN EN ISO 13485 [125], certified by a Notified Body (e.g., TÜV Süd), which itself is accredited as an inspection body according to ISO 17020 [126].

While most routine diagnostic assays are standardized and provided by the IVDR industry, LC-MS/MS methods are occasionally implemented as in-house tests in clinical laboratories. Regulatory requirements for such lab-developed tests vary. In the U.S.A., they fall under the framework of the Clinical Laboratory Improvement Amendments (CLIA). In Germany, they are subject to the Guideline of the German Medical Association on Quality Assurance in Laboratory Medicine (Rili-BAEK). Regardless of the regulatory framework, in-house LC–MS/MS assays represent a substantial challenge, encompassing method development, analytical validation, clinical verification, implementation into routine diagnostic workflows, and continuous maintenance, including quality control, documentation, and ongoing performance monitoring. Fully or semi-automated LC-MS/MS platforms are increasingly available on the market, integrating sample preparation, chromatographic and mass spectrometric separation, partly including LIS connectivity and automated data processing and result evaluation [124].

Beyond its established application to small molecules (endogenous and exogenous compounds) and outside routine clinical laboratory practice, LC-MS/MS is increasingly utilized for the quantification of proteins within research settings and in higher-order reference measurement procedures, for instance in the determination and quantification of HbA_1c_ [127].

In clinical routine diagnostics, analytical performance characteristics (e.g., precision, accuracy, sensitivity and specificity, and measurement range and uncertainty), together with clinical performance and scientific validity, are key determinants of an assay’s overall clinical validity and utility.

## 5. Conclusions and Perspectives

This article reports the results of a literature survey in the PubMed database on biomarkers in clinical medicine research. By nature, the main limitations of the study are its generality, “crudity” and overwhelming number of articles due to the use of “all fields” in the query box of PubMed. Our literature search is not a meta-analysis but rather a critical review and an evaluation of the term biomarker in selected areas of clinical medicine research.

The efforts of generations of scientists on biomarkers resemble the futile labor of Sisyphos. The reasons are multifaceted. First and foremost, the human body is very complex with highly specific yet closely inter-connected organs and with a chemical inventory of thousands of mutually dependent and interwoven LMM and HMM biochemical substances. Secondly, human nature itself. It seems that the ingenious inventions of the human brain cannot solve a problem that is per se unsolvable and far exceed its aptitude. Can the Gordian Knot of biomarkers be loosened in its intact threads?

We have presented and discussed a few examples of biomarkers in this article. There are only a few established and routinely analyzed clinical biomarkers that can be assigned to specific diseases. The majority of these diseases are due to gene deficiency, and measuring the respective biomarkers is less costly than genetic analyses.

ADMA and SDMA are good examples of the need for intense, interdisciplinary and enduring worldwide clinical biomarker research. ADMA and SDMA are seemingly ideal biomarkers. Especially ADMA is undoubtedly involved in many different cardiovascular diseases of adults. Yet, research did not provide convincing evidence of a specific disease or even specific biochemical pathways for ADMA and SDMA. Our analysis indicates that the original and main assumption of ADMA as a potent endogenous inhibitor of NO synthesis in endothelial cells, which still dominates in the scientific literature, has been premature and unfounded. Paradoxically, ADMA and SDMA have been included in the pantheon of biomarkers in humans. Large-scale epidemiological and clinical studies based on the use of validated quantitative LC-MS/MS and GC-MS/MS methodologies provided solid evidence of the involvement of ADMA and SDMA in renal and cardiovascular diseases. Yet, solid scientific evidence of particular biochemical pathways and specific diseases that would classify ADMA and SDMA as clinical biomarkers is still missing. ADMA and SDMA originate from numerous proteins by post-translational arginine dimethylation. The clinical research on the significance of the protein-incorporated ADMA and SDMA and their common precursor MMA for the human body has just started and any conclusion would be premature.

In clinical biomarker research, analytical methods are the alpha and the omega. Everybody knows the dictum: “Progress stands and falls with the quality of analysis”. Many quantitative GC-MS/MS and LC-MS/MS analytical methods have delivered reliable results in the area of biomarker clinical research and helped improve our understanding of the nature of biomarkers. In recent decades, two new MS-based technologies have been introduced in the clinical research of biomarkers: metabolomics and proteomics. They are in the exponential virtually uncontrolled phase of their evolution. At present, these omics approaches based on LC-MS/MS seem to be the Achilles heel [128] rather than stimuli in the discovery and establishment of (new) clinical biomarkers. They generate an enormously high amount of data, suggesting incredibly high numbers of candidate biomarkers and potentially involved interacting pathways. The present situation can be well described by the expressions “not to see the wood/forest for the trees” (British/American) or “den Wald vor lauter Bäume nicht sehen” (German). Such phrases highlight the inability to comprehend the entire situation because of an overemphasis on its parts. It conveys a sense of being overwhelmed by details, leading to a lack of perspective. Statistical and graphical approaches such as Volcano plots and ROC-AUC analysis have been adopted but not properly adapted, without defining “cut-of” values. In PubMed, the use of the combination of the search term proteomics or metabolomics with the term artificial intelligence (AI) biomarkers has already resulted in 441 and 341 articles, respectively. Will artificial intelligence help the human being to overcome an unsolved (unsolvable) problem? Or, perhaps will AI solve the dilemma of disease biomarkers without its creator? Who knows? In our opinion, this is another unachievable expectation, in analogy to the more than 70 biomarkers of oxidative stress [6]. The impact of AI seems to be already unstoppable, and, more importantly, rational scientific and clinical needs seem not to keep pace.

## Figures and Tables

**Table 1:** Number of published articles in PubMed (https://pubmed.ncbi.nlm.nih.gov) found by using the indicated search terms. The right column provides the year of the earliest mention of the respective term. The total number of articles accounts for a good 39 million corresponding to 100% (accessed date, 2 January 2026).

Search Term 1	Number of Articles	Fraction Total (%)	Earliest Date
Human	24,246,879	61.5	1789
Disease	9,249,331	23.5	1781
Health	7,751,989	19.9	1782
Biomarker	1,332,017	3.3	1946
Marker	1,107,265	2.8	1928

**Table 2:** Number (No) of published articles in PubMed found by using the search term 1 *biomarker* and the indicated search term 2 *organ*, year of 1st mention, and fraction of the combined search terms. Bold indicates the highest percentage fraction.

Organ (Term 2)	Articles No of Terms 1 + 2	Year of 1st Mention	Articles No. of Term 2	Fraction of Term 2 (%)
Brain	122,976	1954	2,562,922	4.8
Heart	78,666	1963	1,950,812	4.0
Kidney	70,188	1960	1,123,137	6.2
Liver	94,524	1964	1,429,971	6.6
Lung	88,984	1962	1,209,234	7.4
Stomach	20,819	1967	327,971	6.3
Gut	15,416	1971	218,759	7.0
Bile	8,847	1946	176,701	5.0
Pancreas	12,394	1965	223,829	5.5
**Prostate**	**55,162**	**1972**	**300,726**	**18.3**
Bladder	15,350	1972	234,503	6.6
Eye	19,874	1946	814,641	2.4

**Table 3:** Number (No.) of published articles in PubMed found by using the search term 1 *biomarker* and the indicated search term 2 body fluid or tissue, year of 1st mention, and fraction of the combined search terms. Bold indicates the highest percentage fraction.

Body Fluid/Tissue (Term 2)	Articles No. of Terms 1 + 2	Year of 1st Mention	Articles No. of Term 2	Fraction of Term 2 (%)
Blood	563,344	1947	5,936,706	9.5
Urine	46,012	1959	445,377	10.3
Feces	6465	1957	146,943	4.4
**Saliva**	**9697**	**1959**	**82,801**	**11.7**

**Table 4:** Number of published articles in PubMed found by using the search term 1 *biomarker* and the indicated search term 2 disease or syndrome, year of 1st mention, and fraction of the combined search terms. Bold indicates the highest percentage fraction.

Disease or Syndrome (Term 2)	Articles No. of Terms 1 + 2	Year of 1st Mention	Articles No. of Term 2	Fraction of Term 2 (%)
**Alzheimer**	**34,204**	**1983**	**268,604**	**12.6**
Parkinson	12,402	1971	197,495	6.3
Cancer	551,747	1952	5,510,580	10.0
Diabetes	69,347	1960	1,092,088	6.3
Acromegaly	541	1969	-	-
Hypercholesterolemia	2850	1976	53,246	5.4
Hypertension	30,751	1964	700,592	4.4
Phenylketonuria	264	-	-	-
Citrullinemia	46	1986	800	5.8
Homocysteinemia	767	-	-	-
Peripartum cardiomyopathy	99	2000	1961	5.0
Rheumatism	18,423	1782	328,460	5.6
Acute kidney disease	10,170	1913	95,169	10.7
Chronic kidney disease	18,882	1972	234,446	8.1
Sepsis	15,747	1972	241,554	6.5

**Table 5:** Number of published articles in PubMed found by using the search term 1 *biomarker* and the indicated search term 2 being generally accepted, year of 1st mention, and fraction of the combined search terms. Bold indicates the highest percentage fractions.

Biomarker (Term 2)	Articles No. of Terms 1 + 2	Year of 1st Mention	Articles No. of Term 2	Fraction of Term 2 (%)
Homocysteine Total homocysteine	4336 1619	1979 1990	30,806 8091	14.1 20.0
Alanine aminotransferase (ALT)	8911	1964	70,055	12.7
Aspartate aminotransferase (AST)	7140	1967	57,369	12.5
Oxidative stress	44,503	1987	385,816	11.5
Creatinine	25,970	1967	174,648	14.9
Glucose (blood)	43,246	1962	360,079	11.9
HbA_1c_	10,681	1982	88,270	12.1
Malondialdehyde	10,148	1982	84,187	12.0
Prostaglandin	6167	1973	159,513	3.9
**8-*iso*-Prostaglandin F_2α_**	**933**	**1994**	**2273**	**41.0**
Thromboxane	865	1978	26,248	3.3
Troponin	15,315	1988	41,891	36.8
**NT-proBNP**	**6323**	**1977**	**10,539**	**60.0**
Creatine kinase	6500	1975	45,045	14.4
C-reactive protein	41,533	1975	120,273	34.6
Interleukin 6	24,407	1985	123,326	19.8
Procalcitonin	6039	1991	12,035	50.2
Uric acid (serum)	3277	1981	19,939	16.4
Glutathione	17,278	1964	199,458	8.7
**Prostate-specific antigen**	**35,391**	**1978**	**53,247**	**66.5**
Methyl malonic acid	489	1986	3371	14.5
Chloride	5376	1972	325,130	1.7
BMI	27,302	1989	357,523	7.6
cGMP	876	1976	35,826	2.4
Nitrite	2101	1979	51,036	4.2
Nitrate	2065	1979	100,404	2.1
Cholesterol	28,829	1970	359,019	8.0
LDL Cholesterol	10,316	1980	92,346	11.1
HDL Cholesterol	10,840	1981	86,801	12.4
Lipoprotein (a)	1907	1984	11,269	16.9
Triglycerides	17,926	1971	190,424	9.4
Glomerular filtration rate	11,027	1965	87,335	12.6
Cystatin C	4610	1981	9968	46.1

**Table 6:** Number of published articles in PubMed found by using the search term 1 *biomarker* and the substance class of (**A**) amino acids and (**B**) biogenic amines and polyamines (search term 2), year of 1st mention, and fraction of the combined search terms. The substances are listed in alphabetical order. Bold indicates the highest percentage fractions.

Biomarker (Term 2)	Articles No. of Terms 1 + 2	Year of 1st Mention	Articles No. of Term 2	Fraction of Term 2 (%)
(A) Amino acids				
Alanine	12,280	1964	151,547	8.1
Arginine	6635	1962	150,030	4.4
**Asymmetric dimethylarginine**	**1126**	**1999**	**4066**	**27.6**
**Symmetric dimethylarginine**	**458**	**2003**	**1186**	**38.6**
Asparagine	912	1972	21,144	4.3
Aspartate	12,546	1967	185,423	6.8
**Citrulline**	**2783**	**1986**	**15,824**	**17.6**
Cysteine	7332	1964	169, 944	4.1
Glutamate	8477	1974	198,046	4.3
Glutamine	3155	1980	57,503	5.3
Glycine	4455	1970	123,859	3.6
Histidine	2074	1975	62,352	3.4
Isoleucine	1194	1963	19,308	6.2
Leucine	5037	1971	98,514	5.1
Lysine	4892	1971	118,006	4.2
Methionine	3526	1967	89,005	3.9
Ornithine	1075	1979	23,874	4.6
Phenylalanine	4052	1963	104,858	3.9
Proline	3827	1967	87,050	4.4
Taurine	1584	1984	24,227	6.6
Tryptophan	4025	1961	78,861	5.1
Tyrosine	29,789	1963	356,535	8.3
Valine	2621	1975	39,483	6.6
(B) Biogenic amines and polyamines				
Agmatine	62	1990	1885	3.3
Cystamine	42	1991	2166	1.9
Dimethylamine	161	1973	7625	2.8
Dopamine	5302	1960	191,301	2.8
Histamine	1799	1960	95,705	1.9
Hypusine	14	2000	564	2.5
Putrescine	506	1967	16,834	3.0
Spermidine	496	1973	15,361	3.2
Spermine	428	1973	12,055	3.6
Tryptamine	2689	1964	109,731	2.4
Tyramine	347	1962	10,915	3.2
Catecholamines	6252	1958	296,420	2.1

**Table 7:** Number of published articles in PubMed found by using the search term 1 *biomarker* and various classes of substances (search term 2), year of 1st mention, and fraction of the combined search terms. The substances are listed in alphabetical order. Bold indicates the highest percentage fractions.

Biomarker (Term 2)	Articles No. of Terms 1 + 2	Year of 1st Mention	Articles No. of Term 2	Fraction of Term 2 (%)
**Albumin ratio**	**12,640**	**1975**	**51,160**	**24.6**
Alcohols	26,462	1958	1,145,782	2.2
Aldehydes	9895	1964	174,942	5.7
Amines	25,034	1958	1,154,246	2.2
Amino acids	60,560	1957	1,690,390	3.6
Antioxidants	46,473	1962	824,466	5.6
Bile acids	2627	1946	55,806	4.7
Chemicals	77,136	1960	2,949,241	2.6
DNA	141,384	1961	1,991,502	7.1
Drugs	59,746	1962	2,013,318	3.0
Enzymes	343,162	1947	4,219,843	8.1
Fatty acids	23,774	1961	613,588	3.9
Free radicals	17,377	1964	418,343	4.2
Hormones	115,592	1946	1,885,067	6.2
Ions, metal	906	1982	79,482	1.1
Lipids	100,229	1958	1,759,206	5.7
Lipoproteins	28,180	1967	266,875	10.6
PAH	2847	1985	32,254	8.7
Peptides	302,492	1952	3,276,304	9.2
Pesticides	6881	1956	262,399	2.6
PFAS	378	2005	7596	5.0
Proteins	851,990	1947	9,018,919	9.4
**microRNA** Steroids	**45,079** 55,415	**1972** 1946	**188,472** 1,208,000	**23.9** 4.6
Sugars	26,220	1957	619,879	4.2
Toxins	25,162	1955	521,802	4.8
Vitamins	24,171	1962	501,255	4.8

**Table 8:** Number of published articles in PubMed found by using the search term 1 *biomarker* and post-translational modification (search term 2), year of 1st mention, and fraction of the combined search terms. The post-translational modifications are listed in alphabetical order. Bold indicates the highest percentage fractions.

Biomarker (Term 2)	Articles No. of Terms 1 + 2	Year of 1st Mention	Articles No. of Term 2	Fraction of Term 2 (%)
Acetylation	7683	1957	152,117	5.1
Amidation	18,402	1958	701,812	2.6
Carboxylation	3630	1974	146,619	2.5
**Citrullination**	**2784**	**1986**	**15,828**	**17.7**
**DNA methylation**	**20,399**	**1981**	**113,708**	**17.9**
Glutamylation	5	1996	391	1.3
Glutathionylation	112	1990	2429	4.6
Glycation	10,927	1983	91,187	12.0
Glycosylation	10,280	1976	108,707	9.4
Hydroxylation	1872	1968	123,342	1.5
Hypusination	14	2000	564	2.5
Methylation	33,473	1959	554,524	6.0
**Nitration (tyrosine)**	**1192**	**1991**	**7790**	**15.3**
Oxidation	78,322	1950	1,866,965	4.2
Phosphorylation	29,941	1980	452,386	6.6
Post-translational modification	6721	1981	104,360	6.4
Radiation	43,202	1955	1,185,226	3.6
Reduction	62,059	1946	1,782,255	3.5
Sulfation	9021	1949	292,057	3.1
Sumoylation	348	2004	6699	5.2
Ubiquitination	8745	1982	116,196	7.5

**Table 9:** Number of published articles in PubMed found by using the indicated search terms involving cystatin C, percentage fraction and year of 1st mention.

Search Term	Number of Articles	Fraction (%)	Year of First Mention
CysC	9968	100	1960
CysC biomarker	4610	46.1	1981
CysC biomarker kidney	3125	31.4	1992
CysC biomarker heart	804	8.1	2000
CysC biomarker brain	380	3.8	1988
CysC biomarker liver	317	3.2	1995
CysC chronic kidney disease	2557	25.7	1986
CysC chronic kidney disease biomarker	1156	11.6	1999
CysC acute kidney disease	1591	16.0	1990
CysC acute kidney disease biomarker	1079	10.8	1999
CysC acute kidney injury	1576	15.8	1990
CysC acute kidney injury biomarker	1069	10.7	1998

**Table 10:** Number of published articles in PubMed found by using the indicated search terms, involving cardiovascular disease, percentage fraction and year of 1st mention.

Search Term	Number of Articles	Fraction (%)	Year of First Mention
Cardiovascular disease (CVD)	3,197,818	100	1911
CVD biomarker	153,243	4.8	1962
CVD biomarker troponin	11,794	7.7	1991
CVD biomarker NT-proBNP	6656	4.4	1997
CVD biomarker creatinine	6863	4.5	1969
(a) CVD heart	1,142,551	100	1928
(a1) CVD heart biomarker	53,539	4.6 (a)	1964
(a2) CVD heart troponin	15,483	28.9 (a1)	1975
(b) CVD kidney	178,132	100	1940
(b1) CVD kidney biomarker	15,076	8.4 (b)	1969
(b2) CVD kidney troponin	1366	9.1 (b1)	1993
(c) CVD brain	276,094	100	1945
(c1) CVD brain biomarker	27,791	10.1 (c)	1970
(c2) CVD brain troponin	3660	13.2 (c1)	1985
(d) CVD liver	90,075	100	1945
(d1) CVD liver biomarker	5746	6.4 (d)	1973
(d2) CVD liver troponin	326	5.7 (d1)	1989

**Table 11:** Number of published articles in PubMed found by using the indicated search terms, percentage fraction and year of 1st mention for acute coronary syndrome and takotsubo cardiomyopathy.

Search Term	Number of Articles	Fraction (%)	Year of First Mention
Acute coronary syndrome (ACS)	49,469	100	1946
ACS biomarker	6488	13.1	1988
ACS biomarker troponin	2340	4.7	1994
ACS biomarker BNP	232	0.5	2000
ACS biomarker CK-MB	371	0.7	1996
Takotsubo cardiomyopathy (TTS)	6901	100	2000
TTS biomarker	423	6.1	2002
TTS biomarker troponin	148	2.1	2005
TTS biomarker BNP	24	0.3	2009
TTS biomarker CK-MB	20	0.3	2009

**Table 12:** Number of published articles in PubMed found by using the indicated search terms including diabetes, percentage fraction and year of 1st mention.

Search Term	Number of Articles	Fraction (%)	Year of First Mention
Diabetes	1,094,126	100	1788
Diabetes disease	521,294	47.6	1788
Diabetes mellitus	679,002	62.1	1797
Diabetes type 2	224,211	20.5	1952
Diabetes type 1	98,638	9.0	1946
Diabetes disease biomarker	42,237	100	1968
Diabetes disease biomarker glucose	10,330	23.8	1982
Diabetes disease biomarker HbA_1C_	4436	10.5	1982
Diabetes type 2 C-peptide	4685	100	1978
Diabetes type 2 C-peptide biomarker	539	11.5	1988
Diabetes type 1 C-peptide	4026	100	1972
Diabetes type 1 C-peptide biomarker	484	12.0	1984

**Table 13:** Number of published articles in PubMed found by using the indicated search terms relating to erectile dysfunction, percentage fraction and year of 1st mention.

Search Term	Number of Articles	Fraction (%)	Year of First Mention
Erectile dysfunction (ED)	31,736	100	1945
ED cardiovascular disease	4283	13.5	1946
ED biomarker	872	100	1977
ED biomarker testosterone	163	18.7	1977
ED biomarker prolactin	18	2.1	1998
ED biomarker C-reactive protein	38	4.4	2004
ED biomarker hs C-reactive protein	15	1.7	2006
ED biomarker cholesterol	50	5.7	2003
ED biomarker nitric oxide	95	10.9	2001
ED biomarker cGMP	22	2.5	2001
ED biomarker ADMA	11	1.3	2005
ED biomarker nitrite	4	0.5	2008
ED biomarker nitrate	3	0.3	2008
ED prostaglandin	1116	100	1979
ED prostaglandin E1	1108	99.3	1988
ED prostaglandin E2	13	1.2	1993
ED prostacyclin	26	2.3	1988
ED thromboxane A2	19	1.7	1986
ED biomarker endocan	7	0.8	2017
ED biomarker adropin	3	0.3	2019
ED biomarker fetuin	2	0.2	2015
ED biomarker visfatin	1	0.1	2025

**Table 14:** Results of a PubMed literature search on prostate-specific antigen.

Search Term	Number of Articles	Year of 1st Mention	Year of Onset
Prostate	301,580	1800	1980
Prostate cancer	235,182	1887	1990
Benign prostatic hyperplasia	33,489	1940	1988
Prostate-specific antigen (PSA)	53,394	1960	1990
PSA carcinoma	8232	1975	1984
PSA biopsy	29,385	1975	1990
PSA serum	11,093	1968	1990
PSA immunochemistry	1612	1985	1985
PSA assays	36,570	1969	1990
PSA ELISA	1004	1981	1990
PSA mass spectrometry	519	1995	1995
PSA proteomics	400	2000	2000
PSA metabolomics	124	2010	2010
PSA assays false positive	662	1984	1984
PSA assays false negative	440	1984	1984
PSA overdiagnosis	684	1989	2006

**Table 15:** Number of published articles in PubMed found by using the indicated search terms relating to muscular dystrophies, percentage fraction and year of 1st mention.

Search Term	Number of Articles	Fraction (%)	Year of First Mention
Muscular dystrophy	44,718	100	1902
Muscular dystrophy Duchenne (DMD)	16,455	36.7	1947
Muscular dystrophy Becker (BMD)	2822	6.3	1952
Sclerosis	216,719	100	1874
Multiple sclerosis	124,133	57.0	1879
Amyotrophic lateral sclerosis	39,944	18.4	1897
Focal segmental glomerulosclerosis	9322	4.3	1967
Systemic sclerosis	38,511	17.7	1945
Atherosclerosis	194,526	100	1912
Atherosclerosis biomarker	21,546	11.1	1984

**Table 16:** Number of published articles in PubMed found by using the indicated search terms relating to pregnancy or gestation-associated diseases, percentage fraction and year of 1st mention.

Search Term	Number of Articles	Fraction (%)	Year of First Mention
Pregnancy	1,208,916	100	1781
Pregnancy disease	296,936	22	1781
Pregnancy disease biomarker	12,008	1 (4)	1969
Gestation	1,288,376	100	1781
Gestational disease	318,202	25	1781
Gestational diabetes (mellitus)	38,296	3.2	1945
Gestational disease biomarker	13,115	1 (4)	1969
Gestational diabetes biomarker	2761	0.2 (7)	1972
HELLP syndrome	3614	0.3	1982
HELLP syndrome biomarker	258	(7.1)	1991
Preeclampsia	58,906	4.9	1914
Preeclampsia biomarker	6231	(0.5)	1973
Peripartum cardiomyopathy	1965	0.2	1970
Peripartum cardiomyopathy biomarker	99	(5)	2000
Postpartum cardiomyopathy	992	0.01	1952
Postpartum cardiomyopathy biomarker	47	(4.7)	2000

**Table 17:** Biomarker concentrations in PPCM patients and at 6-month follow-up and in PP controls. Constructed with data reported by Ricke-Hoch et al. [72].

Biomarker	PP-Ctrl	PPCM at Baseline	PPCM at 6 Months
PAI-1 (ng/mL)	21 ± 10	64 ± 38	36 ± 14
C-reactive protein (mg/L)	38 (10–164)	9 (0.5–180)	not determined
hsTNT (ng/mL)	2 (2–6)	19 (9–699)	not determined
NT-proBNP (nM)	59 (30–531)	3087 (175–31,700)	not determined

**Table 18:** Number of published articles in PubMed found by using the indicated search terms relating to post-translational modification, year of 1st mention and year of onset.

Search Term	Number of Articles	Year of 1st Mention	Year of Onset
Post-translational modifications (PTM) proteins	106,391	1946	1982
PTM proteins humans	65,332	1946	1982
PTM proteins human disease	22,630	1950	2003
PTM proteins human disease biomarker	669	1992	2009
PTM humans	67,371	1946	1987
PTM humans proteomics	7594	1997	2004
PTM humans mass spectrometry proteomics (*)	3558	1999	1999
(*) + biomarker	579	2001	2001
(*) + cancer	261	2001	2001
(*) + multiple sclerosis	7	2009	-
(*) + alzheimer	29	2002	-
(*) + parkinson	14	2012	-
(*) + diabetes	27	2007	-
(*) + kidney	15	2004	-
(*) + heart	24	2008	
(*) + liver	34	2001	-
(*) + brain	48	2005	-
(*) + lung	28	2002	-

**Table 19:** Number of articles found in PubMed using the research term human biomarker in combination with the indicated research terms of main analytical methods.

Research	Analytical Method	Articles	Year of 1st Mention	Year of Onset	Articles 2020–2025
Human biomarker	n.a.	1,060,566	1946	1983	269,605
Human biomarker gc	GC	10,988	1976	1992	4079
Human biomarker gc ms	GC-MS	3958	1975	1990	3958
Human biomarker gc ms ms	GC-MS-MS	2354	1985	2002	770
Human biomarker gc ms ms metabolomics	GC Omics	746	2005	2005	287
Human biomarker hplc	HPLC	7938	1977	1983	1196
Human biomarker lc ms	LC-MS	9540	1975	2003	3605
Human biomarker lc ms ms	LC-MS-MS	7992	1988	2003	3006
Human biomarker lc ms ms metabolomics	LC Omics	1815	2002	2010	960
Human biomarker lc ms ms proteomics	Proteomics	2651	2002	2002	812
Human biomarker electrophoresis	Electrophor.	25,551	1947	1986	998
Human biomarker capillary electrophoresis	CE	1448	1975	1992	247
Human biomarker CE metabolomics	CE	178	2007	2010	49
Human biomarker CE proteomics	CE Omics	283	2001	2003	45
Human biomarker RIA	RIA	1237	1975	1981	72
Human biomarker ELISA	ELISA	48,000	1977	1991	10,287
Human biomarker immunochemistry	Immunoch.	67,298	1962	1987	5718
Human biomarker CLIA	CLIA	274	1988	2009	112
Human biomarker artificial intelligence	AI	16,909	1990	2016	12,154

**Table 20:** Comparison between the coverage in HMDB 1.0, 2.0, 3.0 and HMDB 4.0. Taken from [102]. Redrawn with permission.

Category	HMDB 1.0	HMDB 2.0	HMDB 3.0	HMDB 4.0
Total number of metabolites	2180	6408	40153	114 100
Number of detected and quantified metabolites	883	4413	16 714	18 557
Number of detected, not quantified metabolites	1297	1995	2798	3271
Number of expected metabolites	0	0	20 641	82 274
Number of predicted metabolites *	0	0	0	9548
Number of unique synonyms	27 700	43 882	199 668	1 231 398
Number of cmpds with expt. MS/MS spectra	390	799	1249	2265
Number of cmpds with expt. GC/MS spectra	0	279	1220	2544
Number of cmpds with expt. NMR spectra	385	792	1054	1494
Number of cmpds with pred. MS/MS spectra *	0	0	0	98 601
Number of cmpds with pred. GC/MS spectra *	0	0	0	26 880
Number of experimental NMR spectra	765	1580	2032	3840
Number of experimental MS/MS spectra	1180	2397	5776	22 198
Number of experimental GC/MS spectra	0	279	1763	7418
Number of predicted MS/MS spectra *	0	0	0	279 972
Number of predicted GC/MS spectra *	0	0	0	38 277
Number of metabolic pathway maps	26	58	442	25 570
Number of compounds with disease links	862	1002	3105	5498
Number of compounds with concentration data	883	4413	5027	7552
Number of predicted molecular properties	2	2	10	24
Number of metabolite-SNP interactions *	0	0	0	6777
Number of metabolite-drug interactions *	0	0	0	2497
No. of metabolites w. sex/diurnal/age variation *	0	0	0	2901
Number of metabolic reactions *	0	0	0	18192
Number of defined ontology terms *	0	0	0	3150
Number of HMDB data fields	91	102	114	130

## Data Availability

No new data were created or analyzed in this study. Data sharing is not applicable to this article.
